# Gut commensals and their metabolites in health and disease

**DOI:** 10.3389/fmicb.2023.1244293

**Published:** 2023-11-08

**Authors:** Hari Krishnan Krishnamurthy, Michelle Pereira, Jophi Bosco, Jaimee George, Vasanth Jayaraman, Karthik Krishna, Tianhao Wang, Kang Bei, John J. Rajasekaran

**Affiliations:** ^1^Vibrant Sciences LLC., San Carlos, CA, United States; ^2^Vibrant America LLC., San Carlos, CA, United States

**Keywords:** commensals, gut microbiome assessment, gut metabolites, SCFA, gut dysbiosis

## Abstract

**Purpose of review:**

This review comprehensively discusses the role of the gut microbiome and its metabolites in health and disease and sheds light on the importance of a holistic approach in assessing the gut.

**Recent findings:**

The gut microbiome consisting of the bacteriome, mycobiome, archaeome, and virome has a profound effect on human health. Gut dysbiosis which is characterized by perturbations in the microbial population not only results in gastrointestinal (GI) symptoms or conditions but can also give rise to extra-GI manifestations. Gut microorganisms also produce metabolites (short-chain fatty acids, trimethylamine, hydrogen sulfide, methane, and so on) that are important for several interkingdom microbial interactions and functions. They also participate in various host metabolic processes. An alteration in the microbial species can affect their respective metabolite concentrations which can have serious health implications. Effective assessment of the gut microbiome and its metabolites is crucial as it can provide insights into one’s overall health.

**Summary:**

Emerging evidence highlights the role of the gut microbiome and its metabolites in health and disease. As it is implicated in GI as well as extra-GI symptoms, the gut microbiome plays a crucial role in the overall well-being of the host. Effective assessment of the gut microbiome may provide insights into one’s health status leading to more holistic care.

## Highlights

- Gut microbiota-mediated disease progression is not restricted to GI symptoms or GI conditions alone but can also give rise to extra-GI manifestations.
- Gut metabolites, produced by gut microorganisms, are important for host metabolism and interkingdom microbial interaction and are implicated in GI and extra-GI conditions.
- Serum sIgA and IgG levels may help identify commensals that can translocate through the gut and act as pathobionts by activating the immune system.
- Owing to the interkingdom microbial interaction, the various entities comprising the gut microbiome, including the gut bacteriome, mycobiome, archaeome, and virome need to be analyzed together to holistically assess the gut microbiome.
- Gut microbiome assessment has emerged as a clinical tool for a broader understanding of a spectrum of clinical conditions.

## 1. Introduction

The human gastrointestinal (GI) tract harbors a large number of microorganisms which are referred to as the ‘gut microbiome.’ The highly diverse gut microbiome consists of bacteria, fungi, archaea, protozoa, and viruses. The GI tract is a large reservoir of microorganisms with microbial cells outnumbering the host’s cells by a factor of 10 and microbial genes outnumbering the host’s genes by more than 100 times (Bull and Plummer, 2014). Gut residents play various beneficial roles in the host’s metabolism. They aid in the synthesis of nutrients such as vitamins and nonessential amino acids (Vyas and Ranganathan, 2012). They also help in the biotransformation of bile and enable the digestion of nondigestible carbohydrates such as polysaccharides (resistant starches, cellulose, hemicellulose, pectins, and gums), undigested oligosaccharides, unabsorbed sugars and alcohols, and host-derived mucins (Cummings et al., 1987; Koropatkin et al., 2012). Gut bacteria participate in host defenses by physically colonizing niches in the gut which would otherwise be taken up by pathogens. They also strengthen the host’s immunity by participating in the gut-mucosal immune system (Bull and Plummer, 2014). The gut microbiota is also seen to exert effects on the brain via neural, hormonal, and immunological actions giving rise to a communication system called the ‘gut-brain axis’ (GBA) (Bull and Plummer, 2014). As the gut microbiome has crucial functionality and greater complexity than the host’s own karyome, it is now being looked at as a virtual organ or emergent system that can have profound effects on the host (Evans et al., 2013).

Given the crucial role of the gut microbiota in the host’s functions and metabolism, any perturbation to the composition and function of the gut microbiota can have implications on the host’s health. Altered gut microbiota has been associated with chronic diseases ranging from GI conditions to metabolic, neurological, cardiovascular, and respiratory conditions (Durack and Lynch, 2019). A healthy human gut mainly comprises bacterial species belonging to the dominant phyla, *Bacteroidetes* and *Firmicutes* (Shreiner et al., 2015). The other phyla that colonize the gut include *Proteobacteria*, *Actinobacteria*, *Fusobacteria*, *Verrucomicrobia*, and *Lentisphaerae* (Carding et al., 2015). Methanogenic archaea such as *Methanobrevibacter smithii* and *Methanosphaera stadtmanae* are also inhabitants of the gut (Gaci et al., 2014). Additionally, the gut is populated with various fungal species such as *Candida*, *Saccharomyces*, *Penicillium*, and so on (Raimondi et al., 2019). Several populations of pathogenic viruses such as *Enterovirus*, *Rotavirus*, and *Norovirus* are also residents of the human gut (Lecuit and Eloit, 2017). The metabolic functions of these gut microorganisms lead to the production of various gut metabolites such as short-chain fatty acids (SCFA), bile acids (BA), ammonia, phenols, endotoxins, and so on (Wu J. et al., 2021).

Dysbiosis occurring due to the loss of beneficial bacteria, overgrowth of potentially pathogenic microorganisms, or the loss of overall bacterial diversity can lead to chronic GI conditions including irritable bowel syndrome (IBS), functional dyspepsia, and inflammatory bowel diseases (IBD), celiac disease, and colorectal cancer (CRC) (DeGruttola et al., 2016; Singh et al., 2021). Apart from GI illnesses, an altered gut microbiome is also involved in the pathogenesis of non-GI disorders such as allergies, asthma, obesity, non-alcoholic fatty liver disease (NAFLD), cardiovascular diseases (CVD), and neuropsychiatric diseases (Rezasoltani et al., 2020). Additionally, an altered gut microbiome can affect the gut metabolite concentrations which are also implicated in various metabolic conditions (Wu J. et al., 2021). A balance in the gut microbiome is crucial and its imbalance can be used as a clinical tool to understand the development of various diseases. However, given the complexity and dynamic nature of the gut population, studies have not yet come to a consensus on a standard reference that defines a healthy gut. As a result, the comparison between the gut microbiome of healthy and diseased individuals is currently the only means of understanding gut dysbiosis and its related conditions.

This review will shed light on the role of gut micro-organisms including bacteria, archaea, fungi, viruses, and their metabolites in the pathogenesis of GI and extra-GI conditions along with the importance of a holistic approach to assess the gut microbiome.

## 2. Bacteria in the gut

**Figure 1:**
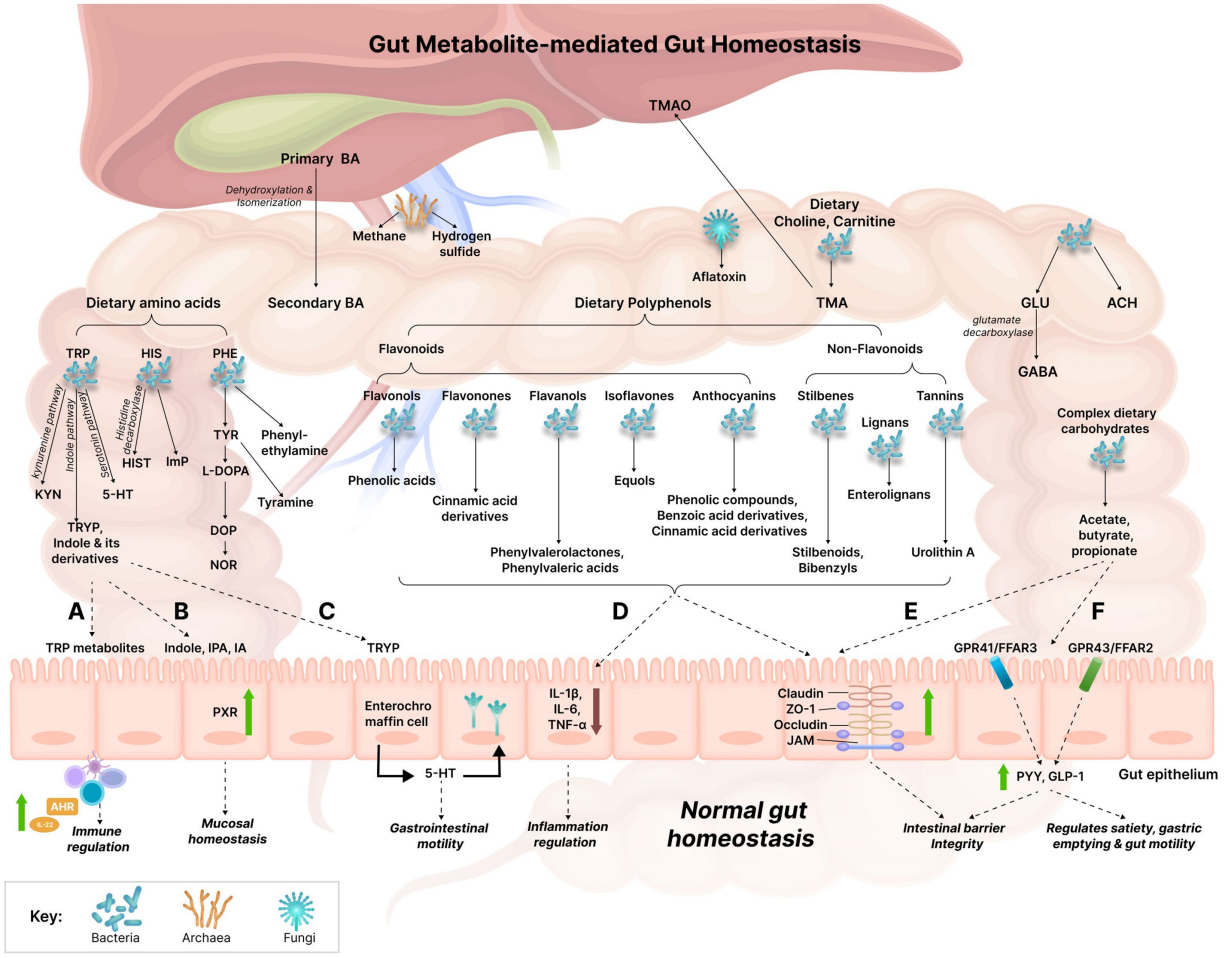
Gut metabolite-mediated gut homeostasis. The gut witnesses’ various metabolic reactions being orchestrated by the gut microbiome which results in the production of numerous metabolites simultaneously. The established effects of several gut metabolites on gut homeostasis have been explained: **(A)** TRP metabolites act on the aryl hydrocarbon receptor (AHR) present in intestinal immune cells, thus affecting innate and adaptive immune responses via AHR activation which results in the increased production of IL-22. **(B)** Indole, IPA, and IA modulate mucosal homeostasis by acting on the pregnane X receptor (PXR); PXR can also downregulate intestinal TNF-α and upregulate tight junction proteins, thereby reducing intestinal inflammation and improving intestinal permeability. **(C)** TRYP induces the release of 5-HT by enterochromaffin cells. 5-HT stimulates gastrointestinal motility by acting on enteric nervous system neurons. **(D)** Polyphenols may reduce gut inflammation by downregulating the expression of pro-inflammatory cytokines (IL-1β, IL-6, and TNF-α). **(E)** Polyphenols and SCFAs can improve gut barrier function by upregulating the expression of the tight junction molecules, such as claudin, zonula occludens-1 (ZO-1), occludin, junctional adhesion molecules (JAM). **(F)** SCFAs bind to the G-protein coupled receptors, GPR41 and GPR43 receptors (also referred to as free fatty acid receptors (FFAR) 3 and FFAR2, respectively) which results in the increased release of the gut hormones, Peptide YY (PYY) and Glucagon-like peptide 1 (GLP-1), thereby increasing intestinal barrier integrity. The increased expression of these hormones also increases satiety and decreases gastric emptying and gut motility. BA, Bile acids; TRP, Tryptophan; KYN, Kynurenine; TRYP, Tryptamine; 5-HT, Serotonin; HIS, Histidine; HIST, Histamine; ImP, Imidazole propionate; PHE, Phenylalanine; TYR, Tyrosine; L-DOPA, Levodopa; DOP, Dopamine; NOR, Norepinephrine; GLU, Glutamate; ACH, Acetylcholine; GABA, Gamma-aminobutyric acid; TMA, Trimethylamine; TMAO, Trimethylamine N-oxide; AHR, Aryl hydrocarbon receptor; IPA, Indole propionic acid; IA, Indole acrylic acid; PXR, Pregnane X receptor; ZO-1, Zonula occludens-1; JAM, Junctional adhesion molecules; GPR, G-protein coupled receptor; FFAR, Free fatty acid receptor; PYY, Peptide YY; GLP-1, Glucagon-like peptide 1; IL, Interleukin; TNF-α, Tumor necrosis factor alpha.

Bacterial colonization begins at birth with the initial gut colonization being instrumental in shaping the composition of the adult’s gut microbiota. An adult human gut is predominantly colonized by *Bacteroidetes* and *Firmicutes* (Carding et al., 2015). The phyla, *Proteobacteria*, *Actinobacteria*, *Fusobacteria*, *Verrucomicrobia*, and *Lentisphaerae* are also present (Carding et al., 2015). Commensal bacteria play crucial roles in human health by contributing to the host gut defense system and helping in various gut metabolic functions (Zhang et al., 2015). They resist the invasion of pathogenic bacteria by competing for nutrients and attachment sites on intestinal mucosal surfaces. This is called “colonization resistance” (Stecher and Hardt, 2008). Another mechanism by which commensal bacteria prevent pathogenic bacteria from colonizing the gut lumen is by reducing intestinal pH via the production of SCFAs which creates an unfavorable condition for pathogenic bacteria colonization (Guarner and Malagelada, 2003). SCFAs such as acetate, butyrate, and propionate are formed from the digestion of complex carbohydrates by colonic bacteria (Figure 1; Zhang et al., 2015). The chemical structures of the SCFAs are given in Supplementary Figure S1. Concentrations of SCFAs depend on microbiota composition, intestinal transit time, host–microbiota metabolic flux of SCFAs, and the fiber content of the host diet. SCFAs are also important for maintaining mucosal immunity and integrity (Rooks and Garrett, 2016). The gut bacteria also prevent pathogen colonization by producing toxic metabolites to inhibit the growth or kill potentially pathogenic bacteria (Beaud et al., 2005).

Gut bacteria benefit the host by aiding in various host processes such as regulating gut motility, producing vitamins, transforming BA and steroids, metabolizing xenobiotic substances, absorbing minerals, and destroying toxins (Macfarlane et al., 2009). SCFAs not only aid in gut immunity but also act as energy sources for the colonic mucosa and help in colonic water absorption and decreases faecal pH (Zhang et al., 2015). Gut bacteria also help in the metabolism and biotransformation of ingested compounds such as lignans and isoflavones (Zhang et al., 2015). They also play a role in host nutrition by synthesizing important nutrients such as vitamin K, thiamine, folate, biotin, riboflavin, and pantothenic acid (Morowitz et al., 2011). Gut bacteria produce secondary BA which appear to activate a number of host nuclear receptors (proteins responsible for sensing BA) even more than host primary BA (Figure 1; Ridlon et al., 2014). Commensal bacteria can alter the metabolic outcome of xenobiotics such as pharmaceuticals, environmental toxicants, and heavy metals by the actions of unique bacterial enzymes that alter the host’s detoxification pathways. They modulate the enzymes or expression of genes involved in xenobiotic metabolism in host tissues. They can also limit xenobiotic absorption in the gut by upregulating the expression of cell–cell adhesion proteins, supporting the protective mucosal layer, and directly sequestering chemicals (Collins and Patterson, 2020). Given the crucial roles of commensal bacteria in host metabolism, gut dysbiosis can lead to serious health implications in the host.

### 2.1. Alteration in commensal bacteria and its association with diseases

Alteration in the commensal population can give rise to various GI and extra-GI conditions. These conditions are discussed below in Table 1. Inappropriate immune responses to gut bacteria can cause chronic intestinal inflammation which is the pathogenesis leading to IBD (Zuo and Ng, 2018). In ulcerative colitis (UC), a type of IBD, intestinal bacterial levels are dysregulated, with reductions in specific *Firmicutes* bacteria (*Faecalibacterium prausnitzii*), and Lactobacilli species and increased *Bacteroidetes* and facultative anaerobes (Zhang et al., 2015; Shen et al., 2018). Intestinal species of the phylum, *Synergistetes* are lower in UC patients (Tang et al., 2021). The population of *Clostridium* spp. is also severely reduced in UC and is accompanied by a reduction in their tryptophan metabolite, indole propionic acid (IPA) (Supplementary Figure S2). Diminished levels of IPA may increase colonic inflammation owing to the inability of IPA to activate pregnane X receptor (PXR), a factor that suppresses intestinal inflammation, thereby contributing to the pathogenesis of UC (Roager and Licht, 2018; Konopelski and Mogilnicka, 2022; Flannigan et al., 2023). The other type of IBD called Crohn’s disease (CD) is believed to be an autoimmune disease in which the body’s immune system attacks the GI tract and causes inflammation. CD is also associated with altered gut microbiota. CD patients are seen to have increased *Ruminococcus gnavus*, *Enterobacteriaceae* (*Klebsiella oxytoca*, *Morganella morganii*, and *Citrobacter amalonaticus*), *Pasteurellaceae* (*Haemophilus* sp.), and *Fusobacteriaceae* and reduced *Erysipelotrichales*, *Bacteroidales*, *Clostridiales*, *Faecalibacterium prausnitzii*, *Bifidobacterium adolescentis*, *Roseburia* spp., and *Veillonellaceae* (*Dialister invisus*) (Joossens et al., 2011; Gevers et al., 2014; Nagao-Kitamoto and Kamada, 2017; Khorsand et al., 2022). A reduction in these commensals is also associated with a decrease in SCFA production which can lead to compromised intestinal barrier integrity and poor protection against inflammation (Silva et al., 2020).

**Table 1:** Disease associated alterations in gut bacterial species.

	Condition	Commensal bacteria	Directionality	Metabolite	Directionality	Reference
GI conditions	Ulcerative colitis	*Bacteroidetes*, facultative anaerobes *Faecalibacterium prausnitzii*, *Lactobacilli* spp., *C. paraputrificum*, *C. sporogenes*, *C. cadvareris*, *P. asscharolyticus*, *P. russellii*, *P. anaerobius*, *P. stomatis*, *Synergistetes*	↑ ↓	Indole propionic acid	↓	Zhang et al. (2015), Roager and Licht (2018), Shen et al. (2018), and Matsuoka and Kanai (2015)
	Crohn’s disease	*Ruminococcus gnavus*, *Klebsiella oxytoca*, *Morganella morganii*, and *Citrobacter amalonaticus*, *Haemophilus* sp., *Fusobacteriaceae*, *Escherichia coli* *Erysipelotrichales*, *Bacteroidales*, *Clostridiales*, *Faecalibacterium prausnitzii*, *Bifidobacterium adolescentis*, *Roseburia* spp., *Dialister invisus*	↑ ↓	Butyrate, propionate, acetate	↓	Joossens et al. (2011), Gevers et al. (2014), Khorsand et al. (2022), Nagao-Kitamoto and Kamada (2017), and Rashed et al. (2022)
	Irritable bowel syndrome	*Veillonella*, *Lactobacillus*, *Ruminococcus*, *Actinobacteria*, *Bacteroidetes*, *Proteobacteria*. *Bifidobacterium*, *Faecalibacterium*, *Erysipelotrichaceae*	↑ ↓	Pyridoxal 5-phosphate Butyrate	↑ ↓	Chong et al. (2019) and Xiao et al. (2021)
Metabolic conditions	Obesity	*Firmicutes*, *Prevotellaceae*, *Veillonella*, *Megasphaera* *Bacteroidetes*, *Odoribacteraceae*, *Clostridaceae*	↑ ↓	Acetate, Propionate, Butyrate	↑	Davis (2016), Kim et al. (2019), and Palmas et al. (2021)
	Diabetes	*Clostridium*, *Bacteroides*, *Veillonella* *Lactobacillus*, *Bifidobacterium*, *Blautia coccoides*/*Eubacterium* rectale group, *Prevotella*	↑ ↓			Murri et al. (2013) and Larsen et al. (2010)
Liver diseases	Non-alcoholic fatty liver disease	*Proteobacteria*, *Enterobacteriaceae*, *Escherichia*	↑			Fukui (2019)
	Cirrhosis	*Faecalibacterium prausnitzii*	↓			Fukui (2019)
	Hepatocellular carcinoma	*Akkermansia muciniphila*, *Ruminococcus*, *Oscillibacter*, *Faecalibacterium prausnitzii*, *Clostridium IV*, *Coprococcus*	↓			Fukui (2019) and Xu et al. (2021)
	Alcoholic-liver disease	*Actinobacteria*, *Proteobacteria* *Bacteroidetes*, *Firmicutes*	↑ ↓	Acetaldehyde, ethanol, ammonia	↑	Malaguarnera et al. (2014)
Cardiovascular diseases	Atherosclerosis	*Collinsella*, *Peptococcaceae*, *Prevotella*, *Firmicutes* *Bacteroidetes*, *Akkermansia muciniphila*	↑ ↓	Trimethylamine	↑	Ahmadmehrabi and Tang (2017), Novakovic et al. (2020), and Liu and Dai (2020)
	Ischemic heart	*Oscillibacter*	↑	Valeric acid	↑	Yamashiro et al. (2017)
Neurological condition	Autism spectrum disorder	*Clostridia*, *Bacteroides vulgatus*, *D. desulfuricans*, *D. fairfieldensis*, *D. piger*, *Caloramator*, *Sarcina*, *Clostridium*, *Alistipes*, *Akkermansia muciniphila*, *Sutterellaceae*, *Enterobacter* *Bifidobacteria*, *Prevotella*, *Coprococcus*, *Dialister*, *Firmicutes*, *Actinobacteria*, *Eubacteriaceae*	↑ ↓	Propionate	↑	Zhang et al. (2015), Ye et al. (2021), and Liu et al. (2019)
Cancers	Colorectal cancer	*Acidaminobacter*, *Phascolarctobacterium*, *Citrobacter farmer*, *Akkermansia muciniphila* *Prevotella*, *Ruminococcus* spp., *Pseudobutyrivibrio ruminis*	↑ ↓	Trimethylamine N-oxide, deoxycholic acid Butyrate	↑ ↓	Zhang et al. (2015), Weir et al. (2013), and Ocvirk et al. (2020)

The intestinal barrier is made up of intercellular binding proteins called tight junction proteins which comprise occludins, claudins, junctional adhesion molecules (JAM), and accessory cytoplasmic proteins such as zonulas occludens (Figure 1). These proteins interact with cytoskeletal proteins and keep the epithelial cells adhered to each other, thereby maintaining the intestinal barrier. *In vitro*, studies have indicated that SCFAs can increase the rapid structuring of the intestinal barrier and can promote the expression of zonulas occludens (ZO-1) and occludins (Figure 1; Caetano and Castelucci, 2022). Particularly, butyrate can increase the expression of tight junction proteins by activating AMP-activated protein kinase (AMPK), via the activation of the G-protein-coupled receptors (GPCR), GPR41 and GPR43, negative regulation of channel formers proteins such as claudin-2, or by inhibiting histone deacetylase (Caetano and Castelucci, 2022). The absence of these effects brought about by lower SCFA levels could be playing a role in the development of CD. In patients with IBS, the abundance of butyrate-producing bacteria is low. As mentioned above, these bacteria generally improve intestinal barrier function, and their decreased levels lead to impairment in intestinal permeability. Animal studies indicated that butyrate reduced inflammation by inhibiting histone deacetylase-1 and inflammatory signaling pathway, NF-κβ, regulating the balance between Th17 lymphocytes (auxiliary T lymphocytes) and Treg lymphocytes (regulatory T lymphocytes), and inhibiting IL-6, signal transducer activator of transcription 3 (STAT-3), and IL-17 (Caetano and Castelucci, 2022). Butyrate can also activate the nociceptive sensory pathways; all these factors, in turn may lead to IBS-related symptoms. IBS patients are seen to have increased growth of *Veillonella*, *Lactobacillus*, *Ruminococcus*, *Actinobacteria*, *Bacteroidetes*, and *Proteobacteria* and decreased growth of *Bifidobacterium*, *Faecalibacterium*, and *Erysipelotrichaceae* (Chong et al., 2019). Additionally, an increase in bacterially synthesized pyridoxal 5-phosphate (Supplementary Figure S5), the active form of vitamin B6 is believed to be involved in the inflammation-mediated pathogenesis of IBS (Xiao et al., 2021).

Altered gut bacterial diversity is an important determinant of susceptibility to metabolic diseases such as obesity and Type 2 diabetes (T2D). Davis (2016) reported that higher caloric intake affected the gut microbiota and was associated with a 20% growth of *Firmicutes* and a 20% reduction in *Bacteroidetes*, which was directly related to the gain in body weight. Thus, obese individuals had higher *Firmicutes* and lower *Bacteroidetes* which also resulted in a high *Firmicutes* to *Bacteroides* ratio (Davis, 2016). SCFAs are also seen to influence host weight with obese individuals having high levels of SCFA (Kim et al., 2019). Elevated SCFA levels may affect body weight and food intake by acting on GPR41 (free fatty acid receptor 3) and GPR43 (free fatty acid receptor 2) receptors. Obese conditions may attenuate the binding of SCFAs to GPCRs leading to increased intestinal energy harvesting and hepatic lipogenesis (Kim et al., 2019). Nevertheless, the exact effect of SCFA on obesity still remains unclear. The occurrence of T2D is also associated with gut microbiota manipulation with *Clostridium*, *Bacteroides*, and *Veillonella* being significantly increased and *Lactobacillus*, *Bifidobacterium*, *Blautia coccoides*/*Eubacterium rectale* group, and *Prevotella* being significantly decreased in diabetic patients (Murri et al., 2013). Moreover, *Bifidobacterium*, *Lactobacillus*, and the *Firmicutes* to *Bacteroidetes* ratio was negatively associated with plasma glucose levels while *Bacteroides-Prevotella* group to *C. coccoide-E. rectale* were positively associated with plasma glucose levels (Larsen et al., 2010; Murri et al., 2013).

Commensal bacteria play an important role in the maintenance of the gut-liver axis. The gut is the primary source of ammonia, as intestinal bacteria decompose protein into ammonia by producing urease which is then transported into the portal vein where it enters the liver and is re-synthesized to urea. This process is called enterohepatic circulation of urea, and it helps maintain low concentrations of ammonia in the blood, as excess ammonia can induce oxidative stress and mitochondrial dysfunction (Campion et al., 2019; Chen Z. et al., 2021). Additionally, gut bacteria metabolize carbohydrates to ethanol which is strongly enhanced under conditions such as obesity, diabetes, or chronic alcohol use. The intestinal oxidation of alcohol (or ethanol) via bacterial alcohol dehydrogenase produces acetaldehyde which can lead to mitochondrial dysfunction and can result in intestinal permeability (Malaguarnera et al., 2014). All these gut endotoxins (for their structures, refer to Supplementary Figure S5) and other luminal bacterial products are subsequently metabolized in the liver which increases the risk of liver diseases. Alcohol consumption affects the composition of the colonic microbiome which indicates that gut dysbiosis may be an important mechanism for alcohol-induced endotoxemia (Mutlu et al., 2009). NAFLD patients had increased *Proteobacteria*, *Enterobacteriaceae*, and *Escherichia* growth (Fukui, 2019). Cirrhosis patients had decreased abundance of the anti-inflammatory commensal, *Faecalibacterium prausnitzii* (Fukui, 2019). Hepatocellular carcinoma (HCC) experienced a reduction in the gut-protective commensal, *Akkermansia muciniphila* and butyrate-producing genera, including *Ruminococcus*, *Oscillibacter*, *Faecalibacterium*, *Clostridium IV*, and *Coprococcus* (Fukui, 2019; Xu et al., 2021). *Actinobacteria* and *Proteobacteria* populations were significantly increased in Alcoholic-liver disease (ALD) and their increase was accompanied by elevated levels of the metabolites, acetaldehyde, ethanol, and ammonia. ALD was also characterized by a reduction in the *Bacteroidetes* and *Firmicutes* species (Malaguarnera et al., 2014).

Atherosclerosis is associated with the increased abundance of *Collinsella*, *Peptococcaceae*, and *Prevotella* (Ahmadmehrabi and Tang, 2017; Liu and Dai, 2020; Novakovic et al., 2020). Additionally, atherosclerosis correlated with the increase in the ratio of *Firmicutes* to *Bacteroidetes* (Liu and Dai, 2020). Many *Firmicutes* species produce the metabolite, trimethylamine (TMA) (Supplementary Figure S5) on metabolizing choline and L-carnitine; TMA is then enzymatically metabolized to trimethylamine N-oxide (TMAO) (Supplementary Figure S5) in the liver (Figure 1). TMAO is seen to contribute to platelet hyperreactivity and thrombosis risk (Ahmadmehrabi and Tang, 2017; Liu and Dai, 2020). As a result, alteration in the TMA-producing bacteria can lead to high plasma TMAO levels which may result in the increased risk of atherosclerosis (Liu and Dai, 2020). Valeric acid (Supplementary Figure S5) produced by *Oscillibacter* was higher in ischemic stroke patients and positively correlated with the level of high sensitivity C-reactive protein (CRP) and white blood cell counts (Yamashiro et al., 2017). The GBA is a potential means for the pathogenesis of the neurological condition, autism spectrum disorder (ASD). The alterations in gut bacteria in ASD have been summarized in Table 1. An overgrowth in the propionate-producing *Clostridia* contributes to ASD pathogenesis. Propionate is seen to worsen ASD-like behavior owing to its ability to cross the blood–brain barrier (BBB) and leading to neuroinflammatory responses and behavioral alterations (Bull and Plummer, 2014; Taniya et al., 2022). The stool samples of CRC patients indicated an increased abundance of *Acidaminobacter*, *Phascolarctobacterium*, *Citrobacter farmer*, and *Akkermansia muciniphila* and a reduced abundance of *Prevotella* (Weir et al., 2013). Moreover, levels of the tumor-protective, butyric acid levels were low in CRC patients, due to the reduced prevalence of the butyrate-producing bacteria including, *Ruminococcus* spp. and *Pseudobutyrivibrio ruminis* (Zhang et al., 2015). Butyric acid may be protective against CRC owing to its tumor suppression properties of acting as a histone deacetylase inhibitor by inducing the cell cycle arrest of cancer cells (Mandal et al., 2001). An increase in the tumor-promoting deoxycholic acid (DCA) was also observed (Ocvirk et al., 2020). DCA is believed to promote tumorigenesis by exerting growth-related actions in a phorbol ester-like manner, by stimulating protein kinase C which is important for the regulation of cellular apoptosis (Milovic et al., 2002).

### 2.2. Alteration in commensal bacteria and its association with gastrointestinal symptoms

The association of gut dysbiosis with various GI and extra-GI conditions suggests that altered gut microbiota could also be affecting GI symptoms and their severities. Here we discuss the role of commensal bacteria in giving rise to GI symptoms (Table 2). Constipation can be associated with altered gut microbiota and there exists a bidirectional relationship between gut microbiota and gut transit time. Gut bacteria can modulate colonic motility, secretion, and absorption; similarly, prolonged colonic transit time caused due to constipation may facilitate the colonization of slow-growing species which can lead to alterations of the gut microecology. The altered bacterial abundance in constipation is given in Table 2. Additionally, elevated levels of SCFAs were observed in the stools of patients suffering from constipation and this increase was associated with the bowel transit time (Zhang S. et al., 2021).

**Table 2:** Gastrointestinal symptom-associated alterations in gut bacterial species.

Symptoms	Commensal bacteria	Directionality	Metabolite	Directionality	Reference
Constipation	*Coprococcus*, *Ruminococcus*, *Blautia*, *Anaerotruncus*, *Clostridium*, *Faecalibacterium* *Bifidobacterium*, *Lactobacillus*, *Bacteroides*, *Prevotella*, *Roseburia*, *Coprococcus 3*	↑ ↓	Butyrate, acetate, propionate	↑	Bull and Plummer (2014) and Zhang et al. (2021)
Diarrhea	*Escherichia*, *Granulicatella*, *Enterobacter cloacae*, *Streptococcus salivarius*, *Streptococcus gallolyticus*, *Blautia*, *Faecalibacterium*, *Lachnospiraceae*, *Ruminococcaceae*, *Enterococcus*, *Granulicatella*, *Proteobacteria*, *Fusobacteria*, *Enterobacteriaceae*, *Pasteurellaceae*, *Lactobacillales*, *Enterobacter*, *Staphylococcus*, *Veillonella*, *Alloprevotella*, *Gammaproteobacteria* *Bacteroides*, *Megasphaera*, *Clostridiales*, *Prevotella*, *Erysipelotrichales, Roseburia*, *Bacteroidaceae*, *Bifidobacteriaceae*, *Ruminococcaecea*, *Lactobacillus*, *Erysipelotrichaceae*, *Holdemanella*, *Subdoligranulum*	↑ ↓	Butyrate, acetate, propionate	↓	Hao and Son-Nam (2022) and Binder (2010)
Bloating	*Proteobacteria*, *Faecalibacterium*, *Actinobacteria*, *Bacteroides uniformis*, *Bifidobacterium adolescentis*	↑ ↓			Noh and Lee (2020)
Flatulence	*Klebsiella pneumonia*, *Proteus*, *E. coli*, *Clostridium*, *Actinobacteria*, *Phascolarctobacterium*, *Bacteroides*, *Coprococcus*, *Blautia*, *Bifidobacteriales*, *Bacteroidetes* (*Parabacteroides*, *Alistipes*, *Bacteroides*), *Firmicutes* (*Enterococcus*, *Dorea*, *Clostridium* spp., *Roseburia intestinalis*, *Ruminococcus*, *Anaerostipes caccae*, *Eubacterium rectale*, *Blautia*, *Veillonella*, *Victivallis vadensis*), *Proteobacteria* (*Desulfotomaculum*, *Desulfovibrio piger*, *Desulfovibrio fairfieldensis*, *Desulfovibrio desulfuricans*, *Desulfobulbus*, *Desulfomicrobium*, *Desulfomonas*, *Fusobacterium* spp). *Oscillospira*, *Ruminococcaceae*, *Bacteroidales*, *Clostridiales*.	↑ ↓	Hydrogen, carbon dioxide, hydrogen sulfide	↑	Manichanh et al. (2014), Mutuyemungu et al. (2023), and Tanagho et al. (2013)
Indigestion	*Prevotella*, *Bifidobacterium*, *Clostridium*, *Proteobacteria*, *Firmicutes*, *Bacteroidetes*, *Fusobacteria*, *Actinobacteria*, *Streptococcus* *Veillonella*, *Actinomyces*	↑ ↓			Tziatzios et al. (2020)

**Figure 2:**
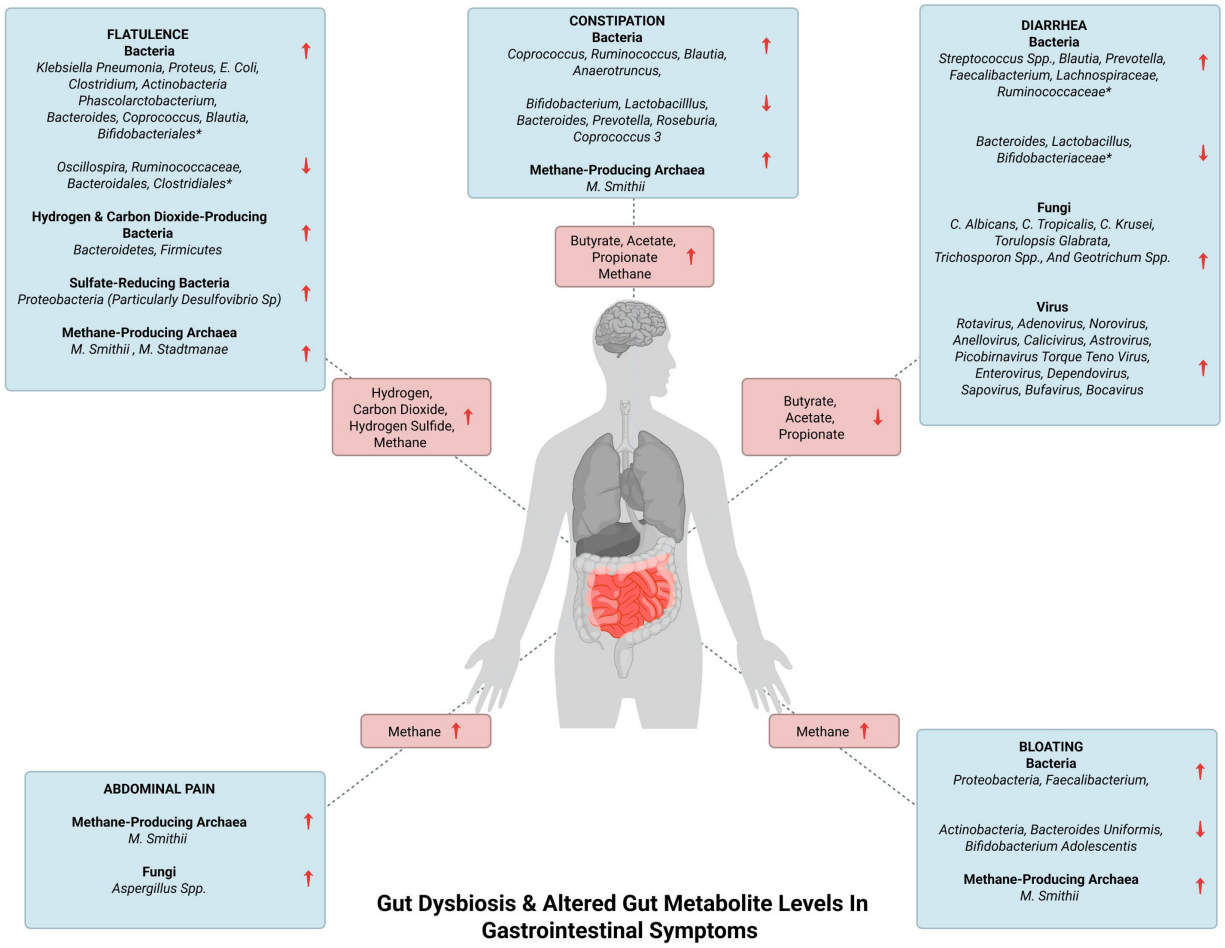
Gut dysbiosis and altered gut metabolite levels in gastrointestinal symptoms. The figure represents gut dysbiosis and the subsequent altered levels of gut metabolites in gastrointestinal symptoms. *The complete list of the increased and decreased commensal bacteria in flatulence and diarrhea is enlisted in Table 2.

Diarrhea, characterized by abnormally loose or watery stools is associated with a reduction in gut taxonomic richness and diversity (Hao and Son-Nam, 2022). Repeated washouts are seen to erode the microbiota, as well as the increased water content in diarrheal stool and lower bowel transit time have been associated with lower diversity in the gut (Hao and Son-Nam, 2022). The alterations in commensal bacteria in association with diarrhea are summarized in Table 2. Reduced SCFA synthesis may aggravate diarrhea while increased production of SCFA can improve the dehydration associated with diarrhea by colonic water reabsorption (Binder, 2010). Increased levels of SCFA in constipation and their reduced levels in diarrhea highlight the fact that the gut ecosystem is in homeostasis when the gut inhabitants and subsequently their metabolites are in optimal levels. Bloating is caused due to increased endogenous gas levels and may be associated with gut dysbiosis, particularly with small intestinal bacterial overgrowth (SIBO). The alterations in gut bacteria that lead to bloating are given in Table 2 (Noh and Lee, 2020). Flatulence is an abdominal symptom that is caused due to the generation of gasses such as hydrogen, carbon dioxide, and hydrogen sulfide (Supplementary Figure S5) in the colon from the fermentation of unabsorbed meal residues by colonic bacteria (Scaldaferri et al., 2013; Manichanh et al., 2014; Mutuyemungu et al., 2023). The common SIBO bacteria, *Klebsiella pneumonia*, *Proteus*, *E. coli*, and *Clostridium* can contribute to flatulence (Tanagho et al., 2013). The alteration in other colonic bacteria associated with flatulence is given in Table 2. These bacterial changes can worsen abdominal symptoms and cause an increase in gas evacuations (Manichanh et al., 2014). Gut dysbiosis can also lead to indigestion and the alteration in bacterial species correlating with indigestion have been highlighted in Table 2. Figure 2 indicates the dysregulation of commensal bacteria and their metabolites in association with GI symptoms.

## 3. Archaea in the gut

Archaea are naturally occurring entities of the gut microbiome. They are unicellular micro-organisms and have been called ‘extreme bacteria’ as they thrive in extreme environments (extreme temperature, pressure, pH) (Rampelotto, 2013). Some archaea have a characteristic metabolism called ‘methanogenesis.’ Methane-producing (methanogenic) archaea are strict anaerobes that generally occur in freshwater and marine sediments, soils, and animal and human guts (Gaci et al., 2014). Methanogenic archaea have been known to be inhabitants of the human gut for more than 30 years through the detection of methane (Supplementary Figure S5) in the breath and isolation of two methanogenic species, *Methanobrevibacter smithii* and *Methanosphaera stadtmanae* (belonging to the order Methanobacteriales). *M. smithii* is the most common methanogenic colonizer in humans followed by *M. stadtmanae. M. smithii* colonizes the GI tract from the cecum to the rectum (Gaci et al., 2014). Methane produced is mainly excreted in flatus while a certain amount is eliminated via the breath.

Owing to their metabolic activities leading to TMA depletion for methanogenesis, archaeal methanogens are believed to have a positive impact on the host’s health. TMA is synthesized by the metabolism of choline, lecithin, L-carnitine, or TMA containing foods (seafood, fish, etc.,) by intestinal microbiota. TMA absorbed in the intestine travels to the liver where it is oxidized to the odorless TMAO which then enters the circulation before elimination via urine (Gaci et al., 2014). As discussed earlier, elevated plasma TMAO levels are associated with the risk of heart conditions (Gaci et al., 2014; Liu and Dai, 2020). Archaeal methanogens, especially of the order, *Methanomassiliicoccales* are likely to metabolize TMA into methane thus, lowering the intestinal TMA concentration. This leads to reduced TMA intestinal absorption and in turn lowers plasma TMAO which can be protective against TMAO-mediated heart conditions (Gaci et al., 2014).

Methanogens are believed to play critical roles in energy harvesting and regulation of glycan digestion. Animal studies have shown that they can affect caloric harvest by increasing the capacity of polysaccharide-eating microorganisms to digest polyfructose-containing glycans, and this action is seen to cause weight gain (Basseri et al., 2012). Furthermore, increased methane produced by methanogens can slow proximal small intestinal transit which can increase weight gain by increasing the total gut microbiome load or the amount of time during which energy is harvested from food. These speculations have been fortified by evidence of obese individuals showing a higher concentration of methane in their breath due to the overgrowth of methanogens (Basseri et al., 2012). Considering the association of archaea with obesity, we believe that gut archaea could also be contributing to other conditions.

### 3.1. Alteration in commensal archaea and its association with diseases

Increased archaeal methane production has also been associated with GI conditions, mainly constipation, and constipation-predominant irritable bowel syndrome IBS (Gaci et al., 2014; Ghoshal et al., 2016). High levels of methane in the colon are believed to be associated with slow bowel transit giving rise to constipation (Pozuelo et al., 2015). However, some studies have observed that IBS patients have a low abundance of methanogens and have attributed this to patients having a decreased ability to remove hydrogen gas from the colon, leading to flatulence or excess gas in the abdomen (Chong et al., 2019). The IBD conditions, CD and UC are associated with an increase in the abundance of *M. stadtmanae* and a reduction in the prevalence of *M. smithii* (Blais Lecours et al., 2014; Ishaq et al., 2016; Ghavami et al., 2018; Rashed et al., 2022). The metabolite, hydrogen sulfide was higher in UC and CD patients. Although there is no mechanistic model that clearly explains this pathogenesis, but with regard to UC, it has been hypothesized that high hydrogen sulfide levels could be damaging the oxidation of butyrate which could lead to impaired barrier function resulting in UC (Guo et al., 2016). *M. stadtmanae* appears to be a strong stimulator of the inflammatory responses in the gut, and thus, its increase contributes to inflammation which is characteristic of IBD (Ishaq et al., 2016). Moreover, a reduced *M. smithii* load can result in the increase of sulfate-reducing bacteria in the gut which can produce toxic hydrogen sulfide which also plays a role in the pathogenesis of IBD (Table 3; Ghavami et al., 2018).

**Table 3:** Disease associated alterations in gut archaeal species.

	Condition	Commensal archaea	Directionality	Metabolite	Directionality	Reference
GI conditions	Ulcerative colitis	*M. stadtmanae* *M. smithii*	↑ ↓	Hydrogen sulfide	↑	Ghavami et al. (2018), Blais Lecours et al. (2014), Rashed et al. (2022), and Ishaq et al. (2016)
	Crohn’s disease	*M. stadtmanae* *M. smithii*	↑ ↓	Hydrogen sulfide	↑	Ghavami et al. (2018), Blais Lecours et al. (2014), Rashed et al. (2022), and Ishaq et al. (2016)
	Irritable bowel syndrome	*M. smithii*	↑	Methane	↑	Ghoshal et al. (2016)
Metabolic conditions	Anorexia	*M. smithii*	↑			Ishaq et al. (2016)
	Obesity	*M. smithii*	↓			Million et al. (2012)
Cardiovascular diseases	Atherosclerosis	*M. smithii*	↑			Duttaroy (2021)
Neurological conditions	Parkinson’s disease	*Methanobrevibacter*	↑			Mohammadzadeh et al. (2022)
Cancer	Colorectal cancer	*Halopelagius* *Methanosphaera*, *Methanococcoides*, *Methanocorpusculum*, *Methanocaldococcus*, *Methanobacterium*	↑ ↓			Mohammadzadeh et al. (2022)

Dysbiosis in the gut archaeome can also lead to various extra-GI conditions (Table 3). Given the critical role of *M. smithii* in harvesting energy, an alteration in its prevalence can have several implications in nutrition and metabolic disorders. The abundance of *M. smithii* was higher in patients suffering from anorexia (Ishaq et al., 2016). Increased *M. smithii* levels in anorexic patients are believed to be an adaptive attempt to optimize energy from low-caloric diets, as *M. smithii* utilizes hydrogen to form methane, allowing for an increase in the transformation of nutrients into calories (Armougom et al., 2009). However, the prevalence of *M. smithii* was lower in the stools of obese patients (Million et al., 2012). This observation seems to be contradictory to the general belief stated earlier, that methanogens and methane are higher in obese individuals (Basseri et al., 2012). As a result, further studies may need to be conducted to understand the role of methanogens in obesity.

As it has been recognized that BA can promote the development of atherosclerosis via bile-salt hydrolase (BSH) and BA receptors, BSH-containing *M. smithii* is believed to contribute to the atherosclerosis (Duttaroy, 2021). The genus, *Methanobrevibacter* was observed to be relatively high in Parkinson’s disease (Mohammadzadeh et al., 2022). CRC is also associated with altered archaeal populations with significantly high levels of the non-methanogenic euryarchaeota, *Halopelagius* and low levels of methanogenic archaea including *Methanosphaera*, *Methanococcoides*, *Methanocorpusculum*, *Methanocaldococcus*, and *Methanobacterium* species (Mohammadzadeh et al., 2022). Reduced levels of methanogenic archaea result in lower conversion of hydrogen to methane resulting in the higher production of hydrogen sulfite (by sulfate-reducing bacteria; Supplementary Figure S5), thereby increasing the potential damage to colonic epithelial cells leading to CRC (Coker et al., 2020). Increased *M. smithii* leading to increased methane production in the gut gives rise to GI symptoms including constipation, bloating, flatulence and abdominal pain (Attaluri et al., 2010; Ghoshal et al., 2016; Mohammadzadeh et al., 2022; Mutuyemungu et al., 2023; Figure 2). *M. stadtmanae* also contributes to the generation of methane in the gut which can lead to flatulence (Scaldaferri et al., 2013; Mutuyemungu et al., 2023; Figure 2).

## 4. Fungi in the gut

Commensal fungi exist in the oral cavity, GI tract, and vagina. The Human Microbiome Project indicated that *Saccharomyces*, *Malassezia*, *Candida*, *Cyberlindnera*, *Penicillium*, *Cladosporium*, *Aspergillus*, *Debaryomyces*, *Pichia*, *Clavispora*, and *Galactomyces* are the most common fungal genera colonizing the gut (Zhang L. et al., 2021). Previously the gut mycobiome received little attention because fungal presence is relatively lower in the gut compared to the bacterial communities. Moreover, fungal analysis was restricted to culture-dependent methods which limited the in-depth understanding of the fungal microbiota. Recent advances in deep-sequencing technologies and bioinformatics analysis have helped in understanding the mycobiome in association with health and disease (Chin et al., 2020).

The gut mycobiome is crucial for host homeostasis. Studies believe that the gut mycobiome is involved in the GBA axis and contributes to gut immunity (Chin et al., 2020). Fungi colonize the mucosal surface of the gut to maintain intestinal homeostasis and systemic immunity (Chin et al., 2020). A study showed that the crosstalk between the gut mycobiome and host immune system can modulate disease outcome, with *Saccharomyces boulardii* (a probiotic yeast) exerting a protective effect against *Clostridium difficile* colitis-induced in mice model. The same study demonstrated that supplementation of *S. boulardii* stimulated the production of intestinal immunoglobulin A (IgA) against *Clostridium difficile* toxin A in mice (Qamar et al., 2001). The mycobiome can be influenced by various factors such as diet, environment, season, comorbidities, and drugs (Wu X. et al., 2021). Fungal populations interact with bacterial species in the gut and can also be affected by the same. Acetic, propionic, and butyric generated by SCFA-producing bacteria can induce transcriptional changes in *C. albicans*, where butyric acid was found to inhibit the yeast-to-hyphal (Y-H) transition, thereby preventing *C. albicans* from colonizing the gut (Sam et al., 2017). In this manner, commensal bacteria and their metabolites keep the mycobiome in check and avoid its pathogenicity.

Similar to bacteria and archaea, fungal metabolism also results in the expression of fungal metabolites which influence host homeostasis and exert biological effects on the host as part of fungi-host interactions (Chin et al., 2020; Wu X. et al., 2021). Fungi produce a diverse array of secondary metabolites which help them thrive in the gut. They produce small molecules like farnesol and fusel alcohols which can act on themselves and regulate their Y-H transition (Sam et al., 2017). For instance, *S. boulardii* produces capric acid which inhibits its Y-H transition, adhesion, and biofilm formation (Sam et al., 2017). The quorum-sensing metabolites, farnesol, and tyrosol produced by *C. albicans* aid in its growth and can also act as virulence factors in the body (Mehmood et al., 2019; Wu X. et al., 2021). Therefore, fungal metabolites can modulate their own pathogenicity. The chemical structures of the mentioned metabolites are given in Supplementary Figure S6.

### 4.1. Alteration in commensal fungi and its association with diseases

Fungal dysbiosis can lead to various GI and extra-GI conditions including cancers, metabolic, heart, liver, and central nervous system (CNS) disorders (Chin et al., 2020; Table 4). The alterations in fungal species in IBD, including UC and CD are given below (Table 4). These conditions are believed to be caused by mucosal inflammation mainly associated with increase in *Aspergillus* and *Candida* species in the gut (Li et al., 2014; Qiu et al., 2017; Rashed et al., 2022). In the gut, the mentioned fungi are recognized by the membrane-bound receptors such as lectin receptors, Toll-like receptors, and scavenger receptor family members on various immunocytes leading to their activation. The activated receptors trigger phagocytosis, respiratory burst, and intracellular signaling pathways, giving rise to the release of multiple pro-inflammatory cytokines. The consequent release of the pro-inflammatory cytokines, IL-Iβ, TNF-α, INF-γ, IL-6, IL-17A, and IL-23 results in the inflammation of the intestinal mucosa which could be the underlying pathogenesis of UC and CD (Li et al., 2014; Qiu et al., 2017). Decreased fungal diversity with the predominant populations of *Saccharomyces cerevisiae*, *C. albicans*, and *Cladosporium* was observed in IBS patients (Botschuijver et al., 2017; Chong et al., 2019). This fungal alteration was associated with visceral hypersensitivity which is believed to be one of the major pathophysiological features of IBS (Matijašić et al., 2020). Animal studies indicate that this hypersensitivity is brought about by intestinal barrier dysfunction followed by the activation of the Dectin-1/Syk pathway which affects the host’s innate anti-fungal immune response which is relevant for visceral hypersensitivity (Botschuijver et al., 2017).

**Table 4:** Disease associated alterations in gut fungal species.

	Condition	Commensal fungi	Directionality	Metabolite	Directionality	Reference
GI	Ulcerative colitis	*Saccharomycetales, Aspergillus, Candida* *Penicillium*	↑ ↓			Qiu et al. (2017)
	Crohn’s disease	*C. neoformans, C. albicans, C. tropicalis, C. glabrata, Cystofilobasidiaceace family, Debaryomyces hansenii* *S. cerevisiae, Malassezia sympodialis*	↑ ↓			Rashed et al. (2022) and Li et al. (2014)
	Irritable bowel syndrome	*S. cerevisiae, Candida, Cladosporium*	↑			Chong et al. (2019) and Botschuijver et al. (2017)
Metabolic conditions	Obesity	*Candida*, *Nakaseomyces*, and *Penicillium* *M. racemosus*, *M. fuscus*	↑ ↓			Matijašić et al. (2020) and Mar Rodríguez et al. (2015)
	Diabetes	*C. albicans*	↑			Gosiewski et al. (2014)
Cardiovascular	Atherosclerosis	*Mucor*	↓			Chacón et al. (2018)
Liver	Alcoholic-liver disease	*Candida* spp	↑			Yang et al. (2017)
	Non-alcoholic steatohepatitis	*Pichia kudriavzevii*, *Candida albicans*, *Candida glabrata*	↑	Ethanol	↑	Mbaye et al. (2022)
Neurological	Rett syndrome	*Candida spp*	↑			Strati et al. (2016)
	Autism spectrum disorder	*Candida, Malassezia, Aspergillus,* and *Penicilliun*	↑			Strati et al. (2017)
	Schizophrenia	*S. cerevisiae C. albicans*	↑			Severance et al. (2012)
Cancer	Colorectal cancer	*Rhodotorula, Malassezia, and Acremonium genera, A. flavus* *S. cerevisiae*	↑ ↓	Aflatoxin	↑	Matijašić et al. (2020)

Metabolic diseases such as obesity are characterized by the increased presence of the genera, *Candida*, *Nakaseomyces*, and *Penicillium* (Mar Rodríguez et al., 2015) while *C. albicans* were highly prevalent in the feces of diabetic patients (Gosiewski et al., 2014). Elevated blood glucose levels in diabetic patients could be creating favorable conditions for intensive fungal colonization leading high to levels of *C. albicans*; however, this claim requires further investigation (Gosiewski et al., 2014). Non-obese individuals have high levels of the genus, *Mucor* (particularly, *M. racemosus and M. fuscus*) indicating that it may be protective against obesity (Matijašić et al., 2020). This protective effect of *Mucor* has been attributed to its chitosan-based wall polysaccharide composition. Animal studies showed that the dietary intake of chitosan was associated with reduced body weight which was achieved by acting on serum leptin and CRP concentrations (Walsh et al., 2013). The abundance of phylum *Zygomycota* consisting of the genus *Mucor* showed negative correlations with carotid intima-media thickness and is thus believed to have protective effects against CVDs (Chacón et al., 2018). Alcoholic patients were observed to have decreased fungal diversity associated with *Candida* overgrowth leading to the risk of ALD (Yang et al., 2017). Fungal overgrowth results in increased fungal products such as β-glucan (fungal cell wall polysaccharides), which can escape from the intestinal lumen via the dysfunctional intestinal barrier to the liver. β-glucan can then induce chronic inflammatory responses in the liver which can progress to ALD (Yang et al., 2017). As fructose consumption is a major risk factor for Non-alcoholic steatohepatitis (NASH), an increase in the ethanol-producing *Candida* spp. which converts fructose to ethanol was observed in NASH patients (Mbaye et al., 2022).

Patients with Rett syndrome (RTT), a progressive neurological disorder characterized by constipation and GI dysfunctions showed an increased prevalence of the genus, *Candida* (Strati et al., 2016). The overgrowth in fungal species associated with ASD and schizophrenia are discussed in Table 4. Fungal ecology is also disturbed in CRC with a characteristic increase in the *Basidiomycota/Ascomycota* ratio. Additionally, *S. cerevisiae* is severely depleted in CRC while the *Rhodotorula, Malassezia,* and *Acremonium* genera and a few *Aspergillus* species are found in abundance. *S. cerevisiae* exhibit regulatory and anti-inflammatory effects in the gut by inducing IL-10 production. A reduction in its levels indicates the importance of its protective effects and may be a factor contributing to CRC (Matijašić et al., 2020). *A. flavus* is increased which results in the elevated levels of its metabolite, aflatoxin (Supplementary Figure S6) which is a carcinogen and is believed to be involved in the pathogenesis of CRC (Matijašić et al., 2020). The fungi, *C. albicans*, *C. tropicalis*, *C. krusei*, *Torulopsis glabrata*, *Trichosporon* spp., and *Geotrichum* spp. can give rise to fungal-mediated diarrhea (Talwar et al., 1990) while increased colonization by *Aspergillus* spp. has been reported to cause abdominal pain (Figure 2; Qiu et al., 2017).

## 5. Viruses in the gut

The gut virome is defined as the viral component of the gut microbiome. It includes eukaryotic viruses and bacteriophages. Eukaryotic viruses can replicate in human cells. Limited studies on the gut virome make it difficult to distinguish the viruses that establish persistent infection and those that can be considered as members of the standard gut microbiota from those responsible for acute infections, particularly for human eukaryotic viruses (Lecuit and Eloit, 2017). Bacteriophages replicate in gut bacteria; thus, their prevalence is modulated by the presence of the host bacteria. Bacteriophages can also lyze bacteria, regulate bacterial contents, and impact relative bacterial counts (Lecuit and Eloit, 2017). Studies state that the human gut phage communities are majorly dominated by DNA phages exhibiting a temperate lifestyle (Lecuit and Eloit, 2017). It is believed that the viral load is approximately similar to that of bacteria in the gut (Minot et al., 2011). Phage can aid in vector transduction (gene transfer) between strains and even bacterial species and can deliver genes encoding toxins, virulence factors, or alternate metabolisms, thus increasing its virulence (Lecuit and Eloit, 2017). The human gut eukaryote viruses are dominated by pathogenic viruses belonging to the genera, *Enterovirus, Rotavirus,* and *Norovirus* (Lecuit and Eloit, 2017). These pathogenic viruses are known to cause transient infections but are also seen to be inhabitants of the gut. The eukaryotic virome appears to be acquired progressively with age, as opposed to bacteriophage richness, which seems greatest in early life and decreases with age (Kapusinszky et al., 2012; Lim et al., 2015).

The gut virome is dominated by bacteriophages with the presence of 10^15^ bacteriophages, outnumbering the commensal bacteria (Matijašić et al., 2020). Bacteriophages are known to play crucial roles in shaping microbial composition, driving bacterial diversity, and facilitating horizontal gene transfer (Sutton and Hill, 2019). They affect the composition and function of the human gut microbiome in both health and disease. Despite their high prevalence and their influence on health, they are the least understood entities of the gut microbiome (Sutton and Hill, 2019). The core phageome in the gut is predominantly populated with dsDNA viruses from the *Caudovirales* order belonging to the families, *Myoviridae*, *Podoviridae*, and *Siphoviridae* and ssDNA viruses of the *Microviridae* family (Manrique et al., 2016). Studies have identified a novel phage, crAssphage, which is believed to be the most prevalent human-associated virus (Dutilh et al., 2014). CrAssphage and novel crAass-like phages are seen to be associated with the bacterial phylum, Bacteroidetes and may become a family within the order, *Caudovirales* (Guerin et al., 2018). Phages generally exhibit a temperate lifestyle with integration into bacterial hosts as prophages. Environmental stressors can activate the lytic cycle leading to viral replication and ultimately the destruction of host cells. Prophages can contribute to genetic factors such as virulence factors or antibiotic resistance genes, thus affecting the dynamics of the gut ecosystem (Matijašić et al., 2020). The phage population in the gut is altered during dysbiosis-related disorders, such as IBD and CRC (García-López et al., 2019).

Research has tried to understand the interplay between the gut virome and the immune system, as bacteriophages and eukaryotic viral particles can activate innate immunity. The detection of viral nucleic acids within cells through several pattern-recognition sensors for RNA (RIG-I and Toll-like receptors TLR7 and TLR8) and DNA (TLR9, cyclic-GMP-AMP) are believed to lead to the expression of type IFN-α and -β, and inflammatory cytokines (IL-1 and IL-6). This activation of innate immunity could protect against pathogenic infections (Duerkop and Hooper, 2013). The predominant presence of phages over eukaryotic viruses suggests that this activation may mainly be driven by phages (Matijašić et al., 2020). However, viruses are also known to have beneficial effects because when viruses were recognized by TLR3 and TLR7, it favored intestinal homeostasis via anti-inflammatory cytokines (Yang et al., 2016).

### 5.1. Alteration in commensal virus and its association with diseases

Although the gut virome may have beneficial effects on the host’s immune system, there is mounting evidence indicating that alteration in the gut virome can lead to intestinal and extra-intestinal conditions. These conditions are summarized in Table 5. The IBD, UC and CD are majorly seen to be associated with the increase in the disease-causing bacteriophages, *Caudovirales* and a reduction in *Microviridae* abundance (Cao et al., 2022). UC patients showed an expansion in the *Caudovirales*, especially *Escherichia* and *Enterobacteria* phage with this expansion being pronounced in the inflamed mucosa compared to the non-inflamed mucosa of these patients (Cao et al., 2022). Murine studies indicated that various *Caudovirales* phage taxa could mediate the TLR9-dependent activation of CD4^+^ T cells which led to intestinal inflammation, via the TLR-dependent production of IFN- γ. This indicates the potential mechanism by which phages activate the gut mucosal immune responses thereby exacerbating inflammation (Cao et al., 2022). As for eukaryotic viruses, the families, *Retroviridae*, *Herpesviridae*, *Hepadnaviridae*, and *Hepeviridae* were relatively higher while *Virgaviridae* was reduced in UC and CD (Rashed et al., 2022). The complete list of the altered abundances of bacteriophages and viruses for both conditions have been enlisted in Table 5. The gut virome had reduced diversity and viral richness in diabetes and their alterations are given in Table 5 (Cao et al., 2022). In T2D, there was an increase in *Siphoviridae*, *Podoviridae*, *Myoviridae*, and unclassified *Caudovirales* families. Synergistic increases in the bacterial families *Enterobacteriaceae* and their predatory phages such as members from *Caudovirales* were also observed in T2D; these concurrent changes in the bacteriome and phageome can be attributed to the “predator–prey” relationship between bacteria and phages (Cao et al., 2022). Adenovirus was seen as a risk factor for NAFLD (Cao et al., 2022). ALD was associated with the increase in eukaryotic viruses including *Parvoviridae* and *Herpesviridae* along with an increase in the phages, *Enterobacteria*, *Escherichia*, and *Enterococcus* (Cao et al., 2022).

**Table 5:** Disease associated alterations in gut viral species.

	Condition	Commensal bacteriophage	Commensal virus	Directionality	Reference
GI conditions	Ulcerative colitis	*Caudovirales* *Microviridae*	*Pneumoviridae*, *Herpesviridae*, *Virgaviridae*, *Circoviridae*, *Picobirnaviridae*, *Orthopneumovirus* *Anelloviridae*, *Polydnaviridae*, *Tymoviridae*, *Coccolithovirus*, *Minivirus*, Vertebrate-infecting virus *Orthopoxvirus*	↑ ↓	Rashed et al. (2022), Matijašić et al. (2020), Cao et al. (2022), and Spencer et al. (2022)
	Crohn’s disease	*Caudovirales*, *Siphoviridae*, *Myoviridae*, *Podoviridae* *Microviridae*	*Retroviridae*, *Herpesviridae*, *Hepadnaviridae*, *Hepeviridae* *Virgaviridae*	↑ ↓	Rashed et al. (2022), Matijašić et al. (2020), and Spencer et al. (2022)
Metabolic conditions	Type 1 diabetes	*Podoviridae*, *Myoviridae*	*Enterovirus*, *Kobuvirus*, *Parechovirus*	↑ ↓	Cao et al. (2022)
	Type 2 diabetes	*Siphoviridae*, *Podoviridae*, *Myoviridae*, *Caudovirales*, *Escherichia phage*, *Geobacillus phage*, *Lactobacillus phage*		↑	Cao et al. (2022)
Liver disease	Non-alcoholic fatty liver disease		*Adenovirus*	↑	Cao et al. (2022)
	Alcohol-associated liver disease	*Enterobacteria phages*, *Escherichia phages*, *Enterococcus phages*	*Parvoviridae*, *Herpesviridae*	↑	Cao et al. (2022)
Cancer	Colorectal cancer	*Inovirus*, *Tunalikevirus* *Enterobacteria phages*, crAssphages	*Herpesviridae*, *Cytomegalovirus*, *Epstein-Bar virus*, *Human papilloma virus*, *Polyomavirus*, *Orthobunyavirus*	↑ ↓	Cao et al. (2022) and Spencer et al. (2022)

The altered gut virome associated with CRC are summarized in Table 5. The increase in *Inovirus* species was reported to regulate the production of bacterial biofilms contributing to the carcinogenesis of the colon. *Epstein–Barr virus* is believed to promote the development of CRC by inducing mutagenesis in intestinal cells. Certain studies indicated that *Polyomavirus* produced T antigen and inactivated key regulatory tumor suppressor proteins, which induced chromosomal instability and malignant transformation of colonic cells (Cao et al., 2022). These factors could be promoting viral-mediated CRC development. Moreover, it is believed that gut phages could also contribute to the pathogenesis of CRC by increasing intestinal permeability, giving rise to a “leaky gut” which facilitates the infiltration of pathogens and triggers chronic inflammation (Tetz and Tetz, 2016). The increase in the viruses, *Rotavirus*, *Adenovirus*, *Norovirus*, *Anellovirus*, *Calicivirus*, *Astrovirus*, *Picobirnavirus Torque Teno virus*, *Enterovirus*, *Dependovirus*, *Sapovirus*, *Bufavirus*, and *Bocavirus* (Figure 2) is seen to give rise to diarrhea (Spencer et al., 2022). Of these, *Rotavirus,* most commonly causes diarrhea followed by *Adenovirus* and *Norovirus*. The mechanism of how these viruses cause diarrhea is unclear, however, *Rotavirus* and *Adenovirus* could be infecting enterocytes of the small intestine resulting in the atrophy of the villi and crypt cell hyperplasia which could lead to fluid malabsorption (Wilhelmi et al., 2003).

## 6. Additional gut metabolites

In addition to the above-mentioned metabolites, there are various bile acid, amino acid, and polyphenol metabolites that are produced by the gut microbiota. The structures of these metabolites are given in Supplementary Figures S1–S6.

### 6.1. Bile acid metabolites

The gut microbiome is a major mediator of BA chemistry and microbially conjugated BA can affect healthy and disease states. Primary BA are synthesized from cholesterol in the liver where they are conjugated to taurine or glycine. Synthesis of primary BA occur via the rate-limiting actions of cholesterol 7α-hydroxylase (CYP7A1) via the classic pathway or the alternative BA pathway which is regulated by sterol-27-hydroxylase (CYP27A1). The classic pathway produces either chenodeoxycholic acid (CDCA) or cholic acid (CA), depending on the activity of sterol 12α-hydroxylase (CYP8B1). The alternative pathway produces mainly CDCA. The expression of CYP7A1 and CYP27A1 is regulated by the gut microbiota. Following food intake, bile is secreted into the duodenum, where it emulsifies and absorbs dietary lipids and fat-soluble vitamins (Figure 1). Upon reaching the colon, BA are subject to extensive metabolism by the gut microbiota (Krautkramer et al., 2021). Several studies have indicated that gut bacteria can modify primary BA by conjugating them to amino acids which give conjugated BA that play important roles in the gut. The predominant amino acid conjugations occur with threonine, glutamic acid, histidine, phenylalanine, tryptophan, tyrosine, lysine, and isoleucine/leucine. Conjugated BA such as threonine-CA, glutamic acid-DCA, and glutamic acid-CDCA can strongly activate PXR. Phenylalanine-CA and tyrosine-CA are strong agonists of the farnesoid X receptor (FXR). PXR and FXR are implicated in immune tolerance (Aleti et al., 2023).

BA also undergo deconjugation by microbial BSHs (removal of glycine and taurine), preventing their active reuptake during enterohepatic circulation. Deconjugated BA then undergo various microbial biotransformations, leading to the formation of numerous secondary BA through dehydroxylation, epimerization and oxidation of hydroxyl groups (Krautkramer et al., 2021). The resultant unconjugated free forms of primary and secondary BA have important roles in the body and their alterations may also be implicated in diseases. Phenylalanocholic acid, tyrosocholic acid, and leucholic acid are formed by the microbiota-mediated conjugation to phenylalanine, tyrosine or leucine by *Clostridium boltae*. DCA and lithocholic acid (LCA) are synthesized by gut microbial 7α/β-dehydroxylation of primary BA (Krautkramer et al., 2021). DCA and LCA are the most potent endogenous ligands of G protein-coupled bile acid receptor 1 (GPBAR1) which is present on cells such as enteroendocrine L-cells of the intestine, Kupffer cells (not on the hepatocyte plasma membrane), cholangiocytes, gallbladder, brown adipose tissue, skeletal muscle, macrophages, and monocytes (Portincasa et al., 2020). Activated PXR, FXR, and GPBAR1 are suppressors of macrophages, dendritic cells and NK-cells which are important for immune tolerance (Aleti et al., 2023). BA also exhibit BBB permeability indicating that they are capable of exerting effects on the CNS. Activation of these receptors in the gut result in the release of fibroblast growth factor 19 (FGF19) and Glucagon-like peptide 1 (GLP-1) which are capable of signaling the CNS (Aleti et al., 2023). GPR131 which is expressed on immune cells (monocytes and macrophages), muscle, spinal cord, adipocytes, and the enteric nervous system, can also be activated by LCA. Animal studies showed that, genetic ablation of GPR131 aggravated intestinal inflammation in colitis disease models (Melhem et al., 2019). Patients with IBD are seen to have altered levels of DCA and LCA (Guzior and Quinn, 2021; Guo et al., 2022). Iso-bile acids are formed by the gut microbial 3α/β-epimerization of primary or secondary BA while allo-bile acids are formed by gut microbial 5β/α-epimerization of primary or secondary BAs. *Clostridium* causes the 7α/β-epimerization of CDCA to yield ursodeoxycholic acid (UDCA). Oxo-BA or keto BA are formed by the microbial oxidation of primary or secondary BA at C3, C7 and C12. The commensals mediating these reactions are summarized in Table 6 (Krautkramer et al., 2021).

**Table 6:** Gut metabolites and their potential association with diseases.

Gut metabolite	Producing organism	Postulated disease association	Reference
Bile acids			
Phenylalanocholic acid, tyrosocholic acid, leucholic acid	*Clostridium boltae*		Krautkramer et al. (2021)
Deoxycholic acid, Lithocholic acid	*Bacteroides*, *Clostridium*, *Escherichia*, *Eubacterium*, *Lactobacillus*	IBD	Guzior and Quinn (2021), Krautkramer et al. (2021), and Guo et al. (2022)
Iso-bile acids	*Eubacterium lentum*, *Clostridium perfringens*, *Ruminococcus gnavus*		Krautkramer et al. (2021)
Allo-bile acids	*Eubacterium*		Krautkramer et al. (2021)
Ursodeoxycholic acid	*Clostridium*		Krautkramer et al. (2021)
Oxo-bile acids or keto-bile acids	*Bacteroides*, *Clostridium*, *Eggerthella*, *Escherichia*, *Eubacterium*, *Peptostreptococcus*, *Ruminococcus*		Krautkramer et al. (2021)
Tryptophan metabolites			
Kynurenine	*Lactobacillus* spp., Putative: *Pseudomonas* spp., *Xanthomonas* spp., *Burkholderia* spp., *Stenotrophomonas* spp., *Shewanella* spp., *Bacillus* spp., members of *Rhodobacteraceae*, *Micrococcaceae*, *Halomonadaceae families*	Depression, schizophrenia, Alzheimer disease, Huntington disease, Type 2 diabetes	Krautkramer et al. (2021) and Cervenka et al. (2017)
Tryptamine	*Ruminococcus gnavus*, *Clostridium sporogenes*	IBS-C	Bhattarai et al. (2018)
Indoxyl sulfate	*Bacteroides* spp	Chronic kidney disease	Krautkramer et al. (2021)
Serotonin	*Clostridia* spp., *Streptococcus* spp., *Enterococcus* spp., *Escherichia* spp., *Lactobacillus plantarum*, *Klebsiella pneumonia*, *Morganella morganii*	IBS	Krautkramer et al. (2021), Barandouzi et al. (2022), and Sikander et al. (2009)
Histidine metabolites			
Histamine	*Morganella morganii*, *Eschericha coli*, *Hafnia alvei*, *Proteus vulgaris*, *Proteus milabilis*, *Enterobacter aerogenes*, *Raoultella planticola*, *Raoultella ornithinolytica*, *Citrobacter freundii*, *Pseudomonas fluorescens*, *Photobacterium damselae*	Asthma	Barcik et al. (2017)
Imidazole propionate (ImP)	*Aerococcus urinae*, *Adlercreutziae equolifaciens*, *Anaerococcus prevotii*, *Brevibacillus laterosporus*, *Eggerthella lenta*, *Lactobacillus paraplantarum*, *Shewanella oneidensis*, *Streptococcus mutans*	Type 2 diabetes	Koh et al. (2018)
Phenylalanine metabolites			
Dopamine	*Enterococcus* spp. (*Enterococcus faecalis*, *Enterococcus faecium*, 77 human isolates of *Enterococcus* spp.), *Lactobacillus brevis*, *Staphylococcus* spp., *Serratia*, *Bacillus*, *Morganella*, *Klebsiella*	Parkinson’s disease, IBD	Krautkramer et al. (2021) and Hamamah et al. (2022)
Norepinephrine	*Lactobacillus*, *Serratia*, *Bacillus*, *Morganella*, *Klebsiella*	Anxiety, depression	Barandouzi et al. (2022)
Phenylacetylglutamine	*Blautia hydrogenotrophica*, *Clostridioides difficile*, *Olsenella uli*, *Romboutsia lituseburensis*	Chronic kidney disease, cardiovascular disease	Krautkramer et al. (2021)
Phenylethylamine	*Staphylococcus*	CD	Chen et al. (2021) and Christian and Berry (2018)
Tyrosine metabolites			
p-Cresol sulfate	*Blautia hydrogenotrophica*, *Clostridioides difficile*, *Olsenella uli*, *Romboutsia lituseburensis*	Chronic kidney disease, cardiovascular disease	Krautkramer et al. (2021)
Tyramine	*Staphylococcus*, *Providencia*, *Lactobacillus bulgaricus*, *Enterococcus faecalis*, *Lactobacillus plantarum*	Hypertension, Ulcerative colitis	Chen et al. (2021), Aydin et al. (2018), and Santoru et al. (2017)
Polyamines			
Putrescine	*Bacteroides* spp., *Fusobacterium* spp	Crohn’s disease Ulcerative colitis	Noack et al. (2000) and Li M. et al. (2022)
Spermidine	*Bacteroides* spp., *Fusobacterium* spp		Noack et al. (2000)
Cadaverine		Crohn’s disease Ulcerative colitis	Li M. et al. (2022)
Other gut amino acid metabolites			
Glutamate	*Lactobacillus plantarum*, *Bacteroides vulgatus*, *Campylobacter jejuni*	Obesity, seizure, autism, and cognition	Chen et al. (2021) and Chang et al. (2020)
GABA	*Bifidobacterium*, *Bacteroides fragilis*, *Parabacteroides*, *Eubacterium*	Anxiety, depression	Chen et al. (2021) and Chang et al. (2020)
Acetylcholine	*Lactobacillus plantarum*, *Bacillus acetylcholine*, *Bacillus subtilis*, *Escherichia coli*, and *Staphylococcus aureus*	Alzheimer’s disease	Chen et al. (2021)

### 6.2. Tryptophan metabolites

Dietary tryptophan can undergo extensive biochemical modifications in the gut, making it an optimal substrate for a variety of transformations. This results in it being an ideal molecule for inter-kingdom communication. Tryptophan also plays a very crucial role in the gut as its metabolites act as ligands of aryl hydrocarbon receptor (AhR) present on T-helper (Th) cells (Monteleone et al., 2011; Figure 1). The binding of these metabolites to AhR mainly initiates the expression of the cytokine, IL-22 which may have inflammatory or protective effects; IL-22 also maintains intestinal microbiota structure (Monteleone et al., 2011). Alterations in tryptophan metabolite levels could affect AhR-driven signals which can differently modulate Th cell response, thereby acting as initiators or attenuators of tissue-damaging T cell– dependent inflammatory processes (Monteleone et al., 2011). This highlights the need for maintaining gut micro-organisms in optimal abundances. Tryptophan, via the host’s mechanisms and gut metabolism, undergoes the kynurenine pathway to produce various chemical intermediates, collectively termed ‘kynurenines’, and NAD+ (Krautkramer et al., 2021). Indoleamine 2,3-dioxygenase 1 (IDO1; in the immune and gut epithelial cells) or tryptophan 2,3-dioxygenase (TDO; in the hepatocytes) bring about the rate limiting conversion of tryptophan to kynurenine (Krautkramer et al., 2021). Gut bacteria, *Lactobacillus* spp., enhance IDO1 expression and IDO1 activity in turn, is believed to regulate microbial community composition (Krautkramer et al., 2021). There are other putative gut inhabitants that are also believed to drive this pathway (Table 6). Kynurenine metabolites can activate GPR35 which regulates mucosal homeostasis and mediates human–microbiota immune tolerance (Aleti et al., 2023). However, in the brain, kynurenine and its intermediates, quinolinic acid and kynurenic acid, were observed to be associated with depression and schizophrenia (Krautkramer et al., 2021). Mice models indicated that the kynurenine pathway was dysregulated in Alzheimer disease (AD) and Huntington disease (Krautkramer et al., 2021). Urinary kynurenic acid were elevated in T2D (Cervenka et al., 2017).

Gut microbial tryptophanase converts tryptophan into indole (Krautkramer et al., 2021). Tryptamine is a tryptophan-derived indole containing monoamine that is also produced in the gut. The commensals, *Ruminococcus gnavus* and *Clostridium sporogenes* express tryptophan decarboxylase, the enzyme responsible for decarboxylation of tryptophan to tryptamine (Bhattarai et al., 2018). Tryptamine is believed to stimulate GI motility via the release of serotonin (5-HT) by the enterochromaffin cells (Figure 1; Aleti et al., 2023). It can activate the serotonin type 3 receptors (5-HT3R) which stimulates gut motility (Aleti et al., 2023). Tryptamine also acts on 5-HT4R to secrete anions and fluid in the colonic mucosa and accelerate GI transit (Li X. et al., 2021). 5-HT4R has been pharmaceutically targeted in diseases associated with slow GI transit such as constipation predominant IBS (IBS-C), owing to its contribution to control intestinal secretion (Bhattarai et al., 2018). As a result, tryptamine, which can modulate 5-HT4R could also be associated with IBS-C and has risen as an attractive therapeutic candidate for the condition (Bhattarai et al., 2018). Tryptamine in the gut can be converted to IPA in the presence of the aromatic amino acid aminotransferase (ArAT) (Li X. et al., 2021); lower IPA levels are implicated in UC (Table 1). IPA can form indole lactate (ILA) via phenyllactate dehydrogenase (fldH) and through dehydration, bacterial species containing phenyllactate dehydratase (fldBC) along with its activator fldI, convert ILA to indole acrylic acid (IA) (Li X. et al., 2021). Indole, IPA, and IA can regulate mucosal homeostasis by acting on the PXR; this indicates the positive role of indole derivatives in maintaining gut homeostasis (Roager and Licht, 2018; Figure 1). Nevertheless, indole can enter the host portal circulation where it gets converted into indoxyl sulfate in the liver (Krautkramer et al., 2021). Indoxyl sulfate is excreted via the kidneys and its high levels have been correlated with the pathogenesis of chronic kidney disease (CKD) (Krautkramer et al., 2021). *Bacteroides* spp. that produced tryptophanase was seen to modulate the levels of indoxyl sulfate in a gnotobiotic mouse model and are suggested to play a role in the target manipulation of the gut microbiota to treat renal diseases (Devlin et al., 2016; Krautkramer et al., 2021).

Serotonin (5-HT) is another important product synthesized from tryptophan by a two-step pathway via the rate-limiting enzyme, tryptophan hydroxylase (TPH). TPH has two isoforms: TPH1 is expressed in enterochromaffin cells within the intestinal mucosa and TPH2 is present neurons of the CNS and the enteric nervous system (Krautkramer et al., 2021). Enterochromaffin cells are responsible for synthesizing more than 90% of total body 5-HT (Krautkramer et al., 2021). The spore-forming bacteria *Clostridia* spp. can induce the transcription of Tph1, subsequently producing 5-HT in the gut (Krautkramer et al., 2021). *Streptococcus* spp., *Enterococcus* spp., *Escherichia* spp., *Lactobacillus plantarum*, *Klebsiella pneumonia*, and *Morganella morganii* can also produce 5-HT (Barandouzi et al., 2022). In the gut, 5-HT acts as a signaling molecule which mainly promotes intestinal motility by acting on multiple receptor subtypes present on enterocytes, smooth muscles and enteric neurons (Sikander et al., 2009). It is particularly seen to exert effects on the enteric nervous system via the activation of the serotonin transporter (SERT) (Bull and Plummer, 2014). Altered 5-HT signaling may lead to both intestinal and extra intestinal symptoms in IBS associated with dysregulated gastrointestinal motility, secretion and sensation (Sikander et al., 2009).

### 6.3. Histidine metabolites

Mammalian and gut bacterial cells can decarboxylate the amino acid, histidine via histidine decarboxylase (HDC) to give histamine (Figure 1). Histamine plays a major regulatory role in the immune system (Krautkramer et al., 2021). The gut bacteria that possess HDC activity leading to the production of histamine are summarized in Table 6. Microbial-derived histamine can regulate immunological responses, visceral nociception, and modulate intestinal motility and gastric acid secretion via the four histamine receptor subtypes (HRH1–4) (Thangam et al., 2018; Aleti et al., 2023). Fecal levels of histamine-secreting bacteria were observed to be high in asthma patients (Barcik et al., 2017). It has been suggested that increased levels of bacterial-derived histamine in asthma patients may contribute to histamine-mediated pathologies due to higher systemic levels of histamine (Barcik et al., 2017). Gut bacteria can also metabolize histidine to imidazole propionate (ImP) via the non-oxidative deamination of histidine to ammonia and urocanate (human metabolite), followed by reduction of urocanate by-urocanate reductase (UrdA). ImP impairs insulin signaling via the activation of the p38γ–p62–mTORC1 pathway and thus, the metabolite was seen to be elevated T2D individuals (Koh et al., 2018).

### 6.4. Phenylalanine metabolites

The catecholamine neurotransmitter, dopamine is mainly produced in the substantia nigra and ventral tegmental areas in the brain. In the gut, the bacteria, *Enterococcus* spp., *Lactobacillus* spp. *Staphylococcus* spp., *Serratia*, *Bacillus*, *Morganella*, and *Klebsiella* can produce dopamine from phenylalanine via a pathway that includes tyrosine, which is converted to levodopa (L-DOPA) by the rate-limiting enzyme, tyrosine hydroxylase (Krautkramer et al., 2021; Figure 1). Gut bacteria synthesize more than 50% of the total dopamine produced by the body (Chen Y. et al., 2021). The gut consists of dopamine and its receptors which regulate gastric secretion, motility, and mucosal blood flow (Chen Y. et al., 2021). Peripheral dopamine cannot cross the BBB (Krautkramer et al., 2021). It regulates movement via dopamine receptors (DRD1-4) and its alteration can have negative effects related to movement (Krautkramer et al., 2021; Aleti et al., 2023). As a result, gut microbial dopamine is also believed to be associated with the pathogenesis and clinical presentations of Parkinson’s disease (Hamamah et al., 2022). Additionally, loss of peripheral dopamine manifests as GI malfunctions, such as delayed gastric emptying and intestinal dysmotility (Hamamah et al., 2022), which explains the possible involvement of an impaired dopaminergic system in IBD (Kurnik-Łucka et al., 2021).

*Lactobacillus*, *Serratia*, *Bacillus*, *Morganella*, and *Klebsiella* can further metabolize dopamine to produce the other catecholamine, norepinephrine (Barandouzi et al., 2022). Gut dysbiosis leading to altered norepinephrine levels underlies the pathophysiology of depressive symptoms and anxiety disorders which are common comorbidities in IBS (Barandouzi et al., 2022). However, whether altered norepinephrine levels are directly implicated in IBD remains to be understood. Commensal bacteria break down phenylalanine to phenylacetic acid (PAA) which can be conjugated to glutamate or glycine in the liver to finally give phenylacetylglutamine (PAGln) (Krautkramer et al., 2021; Zhu et al., 2023). However, the commensals responsible for this reaction are still unclear. PAGln gives rise to the rise of CKD. It has also been associated with overall mortality and CVD in CKD patients with PAGln now being independently identified as a risk factor for major adverse cardiovascular events (Zhu et al., 2023). *Staphylococcus* deconjugates phenylalanine to give phenylethylamine (PEA) which is seen to be elevated in IBD, particularly CD (Christian and Berry, 2018; Chen Y. et al., 2021).

### 6.5. Tyrosine metabolites

Gut bacteria can break down tyrosine to give p-Cresol sulfate (pCS) which leads to the risk of CKD as it is associated with renal damage, inflammation and fibrosis. It is also associated with mortality risk in CVD (Krautkramer et al., 2021). *Staphylococcus*, *Providencia*, *Lactobacillus bulgaricus*, *Enterococcus faecalis*, and *Lactobacillus plantarum* can produce tyramine via the action of aromatic-L-amino acid decarboxylase on tyrosine (Aydin et al., 2018). Tyramine regulates blood pressure, and gut dysbiosis-mediated increase in tyramine could be involved in the pathogenesis of hypertension (Aydin et al., 2018). Tyramine is also known to be an important metabolite responsible for DRD agonism in the gut (Colosimo et al., 2019). A study also showed that fecal tyramine levels were elevated in UC patients; however, mechanism behind this association has not been elucidated (Santoru et al., 2017). Studies in *C. elegans* indicated that commensal *Providencia* can convert tyramine to octopamine in the presence of tyramine β-hydroxylase enzyme produced by the nematode (Chen Y. et al., 2021).

### 6.6. Polyamines

Polyamines are small polycationic molecules that are important for regulating translation, transcription, and cell proliferation and differentiation in both eukaryotic and prokaryotic cells (Tofalo et al., 2019; Nakamura et al., 2021). Various human as well as gut bacterial cells produce polyamines in the body (Tofalo et al., 2019); however, the microbial contribution to polyamine synthesis is still being explored. Putrescine is produced from the decarboxylation of lysine or arginine (Kovács et al., 2019). Spermidine is generated from putrescine by the addition of aminopropyl groups derived from decarboxylated S-adenosyl methionine (Nakamura et al., 2021). Putrescine and spermidine are important metabolites of intestinal bacteria thought to be produced by *Bacteroides* spp. and *Fusobacterium* spp., as indicated by *in vitro* studies (Noack et al., 2000). Cadaverine is produced by bacteria through the decarboxylation of lysine (Kovács et al., 2019). Studies showed that cadaverine could induce HRH4 agonism which is expressed in the GI tract and its altered expression is implicated in IBD and cancer (Colosimo et al., 2019). Fecal putrescine and cadaverine were seen to be significantly higher in CD and UC patients (Li M. et al., 2022); however, mechanism for this increase is still to be understood.

### 6.7. Other gut amino acid metabolites

Intestinal bacteria such as *Lactobacillus plantarum, Bacteroides vulgatus,* and *Campylobacter jejuni* can produce glutamate (Chen Y. et al., 2021). Enteric glutamate plays an important role in transferring intestinal sensory signals to the brain via the vagus nerve; however, enteric glutamate cannot cross the BBB. Alterations in enteric glutamate have been implicated in obesity, seizure, autism, and cognition (Chang et al., 2020). Bacterial glutamate acts as a substrate for γ-aminobutyric acid (GABA) synthesis by decarboxylation with glutamate decarboxylase, found in various bacteria including *Bifidobacterium*, *Bacteroides fragilis*, *Parabacteroides*, and *Eubacterium* (Chang et al., 2020; Chen Y. et al., 2021). Like glutamate, GABA cannot cross the BBB and enteric-GABA serves as a local source of the neurotransmitter where it regulates intestinal fluid and electrolyte transport (Hyland and Cryan, 2010). Enteric GABA is believed to be involved in the regulation of immune cell activity and inflammatory events, as its receptors were found on dendritic cells, macrophages and T-cells, all of them possessing the necessary metabolic machinery for the synthesis and release of GABA (Auteri et al., 2015). It also regulates intestinal barrier stabilization via cholecystokinin (CCK) secreted by enterocytes, enhances the expression of tight junction proteins and increases mucus production by stimulating mucus secreting cells (Aleti et al., 2023). Gut dysbiosis leading to altered GABA levels play an important role in anxiety and depression via GABA receptors (GABAR) (Chen Y. et al., 2021; Aleti et al., 2023). GABA is believed to reduce stress induced by corticosterone via GABAR1-6 (Aleti et al., 2023). Acetylcholine, the common cholinergic neurotransmitter can also be produced by commensal bacteria such as *Lactobacillus plantarum*, *Bacillus acetylcholine*, *Bacillus subtilis*, *Escherichia coli*, and *Staphylococcus aureus* (Chen Y. et al., 2021). In the gut, acetylcholine regulates intestinal motility and secretion and enteric neurotransmission. Alterations in the bacteria producing acetylcholine has been implicated in the pathogenesis of AD (Chen Y. et al., 2021).

N-lactoyl-phenylalanine (Lac-Phe), a peptide conjugate of lactate and phenylalanine is thought to be produced by the condensation of lactate and phenylalanine mediated by the cytosolic enzyme, CNDP2 (Li V. L. et al., 2022). CNDP2+ cells include macrophages/monocytes, other immune cells, epithelial cells, and mesenchymal stem cells localized to diverse organs (Li V. L. et al., 2022). The production of this metabolite is pronounced in blood during and shortly after physical exercise (Hoene et al., 2022). Increased activity-inducible elevations of circulating Lac-Phe levels have been observed in mice, racehorses, and humans, suggesting the metabolite to be a robust molecular effector associated with physical activity across various mammalian species (Li V. L. et al., 2022). Lac-Phe has been proposed to be an ‘exerkine’ as it lowered body weight and adipose tissue mass in obese mice (Hoene et al., 2022). A study conducted in mice indicated that administration of lac-phe for 10 days reduced cumulative food intake, lowered body fat, and improved glucose tolerance, leading to significant weight loss (Li V. L. et al., 2022). Appetite-suppressing effects of Lac-Phe have been attributed to the probable ability to activate the GPCR sensor expressed on CNS neurons involved in appetite regulation (Lund et al., 2022). These findings led to studying the effect of the metabolite in overweight and obese individuals who were subjected to a supervised 8-week long endurance exercise intervention (Hoene et al., 2022). Acute exercise led to a significant increase in Lac-Phe, both before and after the intervention; however, higher levels of Lac-Phe after acute exercise were associated with a greater reduction in abdominal subcutaneous and moderately in visceral adipose tissue during the intervention (Hoene et al., 2022). Elevated levels of Lac-Phe caused greater transient suppression of hunger after each exercise session that contributed to a negative energy balance. With greater understanding on the mechanism behind the metabolite’s effect, Lac-Phe could serve as a biomarker to predict the individual response to exercise-based lifestyle interventions.

### 6.8. Polyphenol metabolites

Polyphenols are a diverse class of secondary plant metabolites obtained from diet. Due to their complex chemical structures (varying from monomers to complex polymers of high molecular weight), they are poorly absorbed in the small intestine. As a result, they reach the colon where they can influence as well as get metabolized by resident microbiota. Polyphenols can stimulate several of keystone bacterial species such as *Akkermansia muciniphila*, *Bacteroides thetaiotaomicrom*, *Faecalibacterium prausnitzii*, *Bifidobacteria*, and *Lactobacilli* (Rodríguez-Daza et al., 2021). In turn, these unabsorbed polyphenols get metabolized by intestinal microbiota into bioactive, low-molecular-weight phenolic metabolites which are easily absorbed by the colon (Mithul Aravind et al., 2021). This polyphenol digestion action exerts prebiotic effect largely associated with promoting the growth of beneficial commensals or suppression of pathogenic bacteria. Thus, polyphenols help in preventing infections in the gut, along with other health benefits including improved stool quality and reduced risk of gastroenteritis, colon cancer, IBD and other related infection, and metabolic conditions (Mithul Aravind et al., 2021). Moreover, polyphenols can reduce gut inflammation by reducing the expression of proinflammatory cytokines and improve intestinal barrier function by upregulating the expression of tight junction molecules (Caban and Lewandowska, 2022; Figure 1).

Polyphenols are broadly classified as: flavonoids and non-flavonoids. The subgroups of flavonoids include flavonols, flavonones, flavanones, flavanols, isoflavons, and anthocyanins while that of non-flavonoids include phenolic acids, stilbenes, lignans, and tannins (Mithul Aravind et al., 2021; Caban and Lewandowska, 2022). The flavonol, quercetin and the flavonone, luteolin get degraded by *Eubacterium ramulus*, to give 3,4-dihydroxyphenylacetic acid (3,4HPAA) (phenolic acid) and 3-(3,4-dihydroxyphenyl) propionic acid (cinnamic acid derivative), respectively (Braune et al., 2001). Studies showed that 3,4HPAA prevented malignant transformation and mitochondrial dysfunction in CRC cell lines (Catalán et al., 2020). Intestinal microbiota including *Actinobacteria* and *Clostridium* clusters have the metabolic capacity to perform glycosidic linkages, C-ring fission and the degradation of the heterocyclic structures of the flavanols, catechins. This results in the formation of smaller molecules including phenylvalerolactones and phenylvaleric acids (Li Q. et al., 2021; Swer et al., 2023). Isoflavones such as daidzein are metabolized by the gut bacteria, *Adlercreutzia equolifaciens*, *Asaccharobacter celatus*, *Enterorhabdus mucosicola*, *Slackia isoflavoniconvertens*, *Slackia equolifaciens*, *Bifidobacterium* spp., and *Lactococcus* spp. *to produce* equols (EQs) (Mayo et al., 2019). EQs can cross the BBB and reduce the production of pro-inflammatory cytokines, preventing neuroinflammation. They are also known to protect microglia against oxidative stress, prevent neural apoptosis, and induce neural generation (Swer et al., 2023). Anthocyanins are also microbial-derived metabolites obtained from dietary sources such as berries, grapes, plums, and other foods containing high natural colorants. They can be metabolized by commensals such as *Bifidobacterium* and *Lactobacillus* species (Swer et al., 2023). Bacterial degradation of anthocyanins yields phenolic acids such as vanillic acid and syringic acid, benzoic acid derivatives including, 4-hydroxybenzoic acid, as well as other catabolites such as catechol, pyrogallol, resorcinol, tyrosol, 3-(3′-hydroxyphenyl) propionic acid, dihydrocaffeic acid and 3-(4′-hydroxyphenyl) lactic acid (Tian et al., 2019). Anthocyanins exhibit antioxidant and neuroprotective effects. They can reduce neuroinflammation and regulate cell signaling pathways (Swer et al., 2023). Korean black bean anthocyanin was able to reduce neural apoptosis in an APP/PS1 transgenic mouse model of AD (Ali et al., 2018).

*Slackia equolifaciens* and *Adlercreutzia equolifaciens* convert the stilbenes, resveratrol to dihydroresveratrol (DHR) (stilbenoid) through hydrogenation (Wang et al., 2022). Resveratrol can also be converted to 3,4-dihydroxy-trans-stilbene and 3,4-dihydroxybibenzyl (lunularin), however, the exact bacteria mediating these actions are still unknown (Wang et al., 2022). Lignans such as secoisolariciresinol diglucoside and pinoresinol diglucoside derived from flax seeds can be metabolitzed by *Ruminococcus* spp. in humans, to give the enterolignans, (+)-dihydroxyenterodiol and (+)-enterolactone (Senizza et al., 2020). Enterolactone is believed to have anti-tumor properties as it inhibited the growth of prostate cancer cell lines *in vitro* and *in vivo*, through a caspase-dependent pathway (Senizza et al., 2020). Ellagitannins and ellagic acid of the sub-class tannins are metabolized mainly by *Clostridium* spp. and *Eubacterium* spp. to yield Urolithin A (Mithul Aravind et al., 2021). A study in mice indicated that Urolithin A was able to alleviate AD symptoms and improve cognitive functions, inhibit neural apoptosis, induce neurogenesis, and reduce the pro-inflammatory cytokines IL-1β and TNF-α in the cortex and hippocampus (Gong et al., 2019). Owing to the antioxidant, anti-inflammatory, and neuro-protective activities of polyphenol metabolites on various aspects of the gut and the body at large, polyphenols are arising as therapeutics for GI and extra GI ailments. The metabolism of the sub-groups of polyphenols to their respective metabolite class have been represented in Figure 1.

## 7. Unexplored gut metabolites

In this section we discuss the metabolites that have recently been explored in the field of gut metabolism.

### 7.1. N-acyl amino acids

N-acyl amino acids (NAAs) are endogenous signaling molecules that have an amide bond which covalently links an amino acid to the acyl moiety of a long-chain fatty acid (Battista et al., 2019). NAAs are believed to play key roles in lipid signaling and have been recognized as potential ligands, engaging the novel binding sites of membrane proteins such as GPCRs (Battista et al., 2019). NAAs can be produced in bacterial membranes under stressful conditions; however, the significance of their roles remain unclear (Battista et al., 2019). *E. coli* can produce N-acyltyrosines as metabolites which exhibit antibiotic activity against *Bacillus subtilis* and moderately inhibit the potential of *Pseudomonas aeruginosa* to form a biofilm (Arul Prakash and Kamlekar, 2021). Various Gram-negative bacteria synthesize N-acyl homoserine lactone products from N-acyl homoserine lactone synthases which are used as quorum-sensing signaling molecules (Craig et al., 2011). GPCRs are considered to be crucial mediators of host–microbial interactions in the human microbiome. Studies suggest that several GI bacteria express N-acyl amide synthase genes and their products are believed to interact with GPCRs that regulate GI physiology (Burstein, 2018). N-acyl-3-hydroxypalmitoyl-glycine (also known as commendamide) is produced by *Bacteroides vulgatus* via the N-acylation of glycine with a β-hydroxy fatty acid via N-acyltransferase activity (encoded by the *glsB* gene). This is followed by *O*-acylation of the free hydroxyl with a secondary fatty acid via an *O*-acyltransferase (encoded by *glsA*) to produce a diacylated amino acid lipid. This molecule resembles the long-chain N-acyl-amides that function as mammalian signaling molecules through activation of GPR132, resulting in the interaction with the host’s immune system (Lynch et al., 2017). Various NAAs have been detected in *E. coli* and in Gram positive bacteria with C_12_ to C_17_ fatty acyl moieties (Hueber et al., 2022). Microbial-NAAs may affect GI physiology. Studies believe that *Gemella* spp. may encode N-acyl serinols that are closely associated with the small intestinal mucosa, suggesting this site to be potentially important for N-acyl-amide-mediated interactions (Cohen et al., 2017).

Generally, the GPCRs with which bacterial N-acyl amides are found to interact collectively come under ‘lipid-like’ GPCR gene family. The significance of this GPCR family for the regulation of host–microbial interactions is highlighted by their presence in the GI track which are enriched in bacteria that are predicted to synthesize GPCR ligands. Lipid-like GPCRs may have roles in disease models that are correlated with alterations in microbial ecology. Mice model studies indicated that N-palmitoylserinol which activates GPR119 regulating glucose homeostasis is associated with obesity and diabetes (Cohen et al., 2017). N-acyl serinol is also an agonist of GPR119. N-acyl alanine and N-acyl-3-hydroxypalmitoyl-glycine can activate GPR132 which is expressed on immune cells such as dendritic and NK-cells, and to a lesser extent on B-cells, macrophages and brain regions such as midbrain and corpus callosum (Aleti et al., 2023). N-acyl lysine/ornithine promotes the action of sphingosine-1-phosphate receptor 4 (S1PR4) expressed on most of the immune cells (Aleti et al., 2023). Prostaglandin I2 receptor (PTGIR) expressed selectively on monocytes and dendritic cells is seen to be antagonized by N-acyl glutamine. Similarly, prostaglandin E receptor 4 (PTGER4) which is expressed on immune and intestinal cells, and which regulates the secretion of GLP-1 is antagonized by N-acyl serinol, N-acyl alanine, N-acyl lysine/ornithine, and N-acyl glutamine. The mentioned GPCRs are known to regulate a wide range of biological processes such as immune cell differentiation (S1PR4, PTGIR, PTGER4), immune cell trafficking (S1PR4, GPR132), and tissue repair (PTGIR). Moreover, various disease models have highlighted the implications of these lipid-like GPCRs in the pathophysiology of conditions such as obesity and diabetes (GPR119), colitis (S1PR4, PTGER4, PTGIR), autoimmunity (GPR132) and atherosclerosis (GPR132, PTGIR) (Aleti et al., 2023). The structures of all the mentioned NAAs are given in Supplementary Figures S6, S7.

With various combinations of amine head groups and acyl tails, NAAs represent a large and functionally diverse class of microbiome-encoded GPCR-active signaling molecules. Current strategies for treating dysbiosis-related conditions such as IBD or diabetes are failing to address dysfunction of the host–microbial interactions that are likely to be part of the disease pathogenesis. Engineering bacteria to deliver bioactive small molecules produced by the gut microbiome may help address diseases of the microbiome by modulating the native distribution and abundance of crucial metabolites. As GPCRs have been extensively validated as therapeutic targets, modulating GPCRs by microbiota-derived NAAs can potentially be used as a therapeutic strategy for the treatment of human diseases (Cohen et al., 2017).

### 7.2. Colibactin

Colibactin is a genotoxic gut metabolite produced by a hybrid non-ribosomal peptide synthetase–polyketide synthase assembly line encoded by the *pks* genomic island (also known as *clb* gene cluster). *pks* genomic islands are present in *Enterobacteriaceae* that belong to the phylogenetic group B (Mousa, 2022). The structure of colibactin is given in Supplementary Figure S7. Various strains of *pks + E. coli* are seen to produce the metabolite. Although the *pks^+^ E. coli* ‘s genotoxicity and its ability to cause DNA double-strand breaks in cultured epithelial cells was identified a decade ago, the chemical species responsible for these effects wasn’t identified. This was because the isolation and structural elucidation of colibactin was challenged due to its contact-dependent synthesis, minimal expression, and chemical instability (Mousa, 2022). Metabolomic studies identified that colibactin was contributing to *pks^+^ E. coli’*s genotoxic effects with its ability to cause double-strand DNA breaks, eukaryotic cell cycle arrest, and chromosome aberrations (Mousa, 2022; Silpe et al., 2022). These studies suggested that colibactin has a pseudodimeric structure, with a reactive cyclopropane warhead at each end which could account for its characteristic DNA-alkylating ability (Silpe et al., 2022). Colibactin–DNA adducts have been identified in mammalian cells and in mice (Silpe et al., 2022). Mouse models indicated that colibactin- *pks + E. coli* could promote colon tumorigenesis leading to CRC (Silpe et al., 2022). DNA alkylation is mediated by the electrophilic cyclopropane moiety of colibactin which leads to the formation of the covalent bond between colibactin’s electrophilic warhead (azospiro 2,4 bicyclic ring) and the nucleophilic DNA yielding a colibactin–DNA adduct. This creates a second electrophilic center that further reacts with DNA to form a crosslink. Cross-linked DNA adducts trigger multiple DNA repair signaling pathways which result in the formation of double-stranded breaks and further, carcinogenesis (Mousa, 2022).

Colibactin-producing *E. coli* was over-represented in the gut microbiome of patients with IBD (Dubinsky et al., 2020). The interplay between colibactin and inflammation is unclear. It is believed that the colibactin may alter the gut microbial composition which might promote the abundance of pro-inflammatory bacteria. A study which observed a shift in gut microorganisms during inflammation (with unknown directionality of DNA damage leading to tumor and inflammatory cytokines and cells promoting tumourgenesis by creating a microenvironment that enables more DNA damage) was characterized by enrichment in genera of *E. coli.* This microbial shift could be associated with inflammation (Mousa, 2022). A cell-line study revealed that the expression of mucin genes which form the mucous layer on the intestinal epithelia decreased the genotoxic effect of colibactin. As inflammation is associated with degradation of the mucous layer and a reduction in tight junction proteins, also known as “leaky gut,” a leaky gut could be an important factor that causes colibactin-mediated inflammation which could further lead to cancer (Mousa, 2022). The recent understanding of *E. coli* and other commensals belonging to *Enterobacteriaceae* producing a genotoxic metabolite highlights the concept that other commensal inhabitants could be doing the same. Microbiome chemistry is a relatively uncharted area and we are still to understand the cryptic genes and encoded chemistry of the microbiome we host. Investigating the scope of commensals producing toxic metabolites may arise as an interesting niche to explore.

## 8. Interkingdom interactions

**Figure 3:**
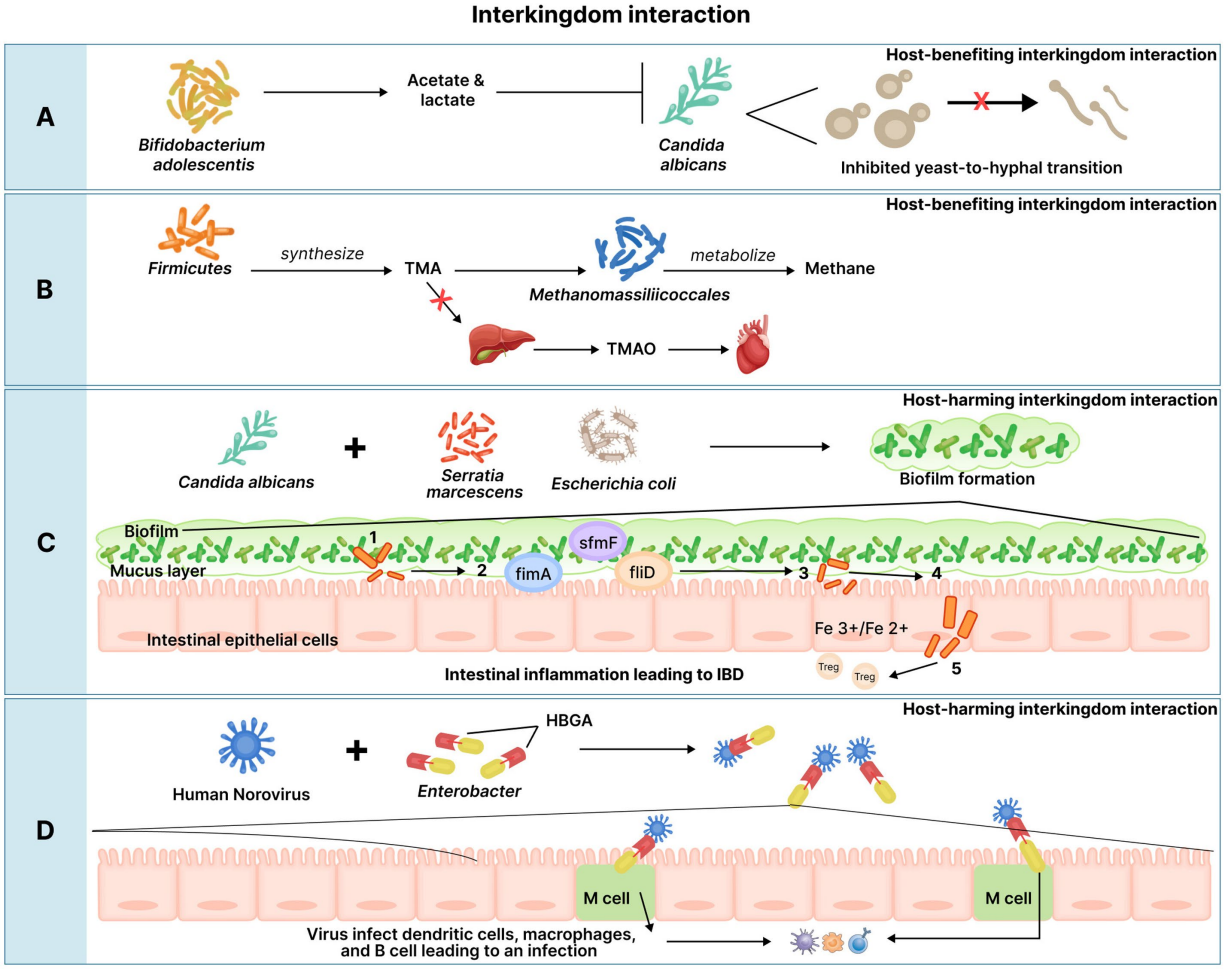
Interkingdom interactions. Interkingdom interactions can have beneficial or harmful effects on the host. **(A)** The *Bifidobacteriaceae* family, *Bifidobacterium adolescentis* in particular, produce acetate and lactate that exhibit antagonistic activity against *C. albicans* by inhibiting its yeast-to-hyphal transition. **(B)** *Firmicutes* species produce the metabolite, TMA on metabolizing choline and L-carnitine; TMA can then be enzymatically metabolized to TMAO in the liver, which in high circulating levels is associated with thrombosis risk. *Methanomassiliicoccales* are likely to metabolize TMA into methane which results in lower levels of intestinal TMA available for hepatic conversion to TMAO. **(C)** The fungi, *C. albicans* and the two bacterial species, *Serratia marcescens* and *Escherichia coli* can interact to form a mixed biofilm in IBD; this interaction is believed to aid in the formation of a thicker mixed biofilm than any of the species could generate individually. The mixed biofilm is postulated to affect the intestinal lumen in the following manner: Fragments of the formed microbiota biofilm cross the mucus barrier and adhere to the epithelial surface. The microbiota may release virulence factors (*E. coli* may realease Hemolysin) and express genes involved in epithelial adhesion (e.g., fimA, sfmF, and fliD). Iron-uptake from the gut micro-environment enables the transformation of gut commensals to pathobionts, helping in the expression of virulence factors. Pathobionts then translocate through the epithelium paracellularly and transcellularly. Translocation of pathobionts through the enterocytes activate proinflammatory pathways in the host via the activation of regulatory T cells which suppress protective responses to inflammatory stimuli, resulting in inflammation in IBD. **(D)** HNoV bind to HBGA present on commensal bacteria such as *Enterobacter* sp. which are then are then transcytosed across the intestinal epithelium via M cells. Following transcytosis, HNoVs infect dendritic cells, macrophages, and B cells leading to HNoV infection. TMA, Trimethylamine; TMAO, trimethylamine N-oxide; IBD, Inflammatory Bowel Disease; HNoV, Human norovirus; HBGA, histo-blood group antigens.

The gut microbiome consists of the bacteriome, mycobiome, archaeome, and virome (Willis et al., 2019). As a part of the gut micro-ecosystem, these microorganisms are seen to interact with each other across kingdoms. Interkingdom interactions can be synergistic, for example, cooperating for mutual growth, or antagonistic, for instance, competing for niche colonization and nutrient sources (Lapiere and Richard, 2022). These interactions can in turn, be beneficial or harmful to the host (Figure 3). Studies have demonstrated the impact of metabolite-driven interkingdom communication on human health (Lapiere and Richard, 2022). Bacteria in the microbiome keep fungi in check by producing extracellular substances that inhibit the growth or the Y-H transition of pathobionts, such as *C. albicans*. Members of the *Bifidobacteriaceae* family, particularly *B. adolescentis*, produced acetate and lactate (Supplementary Figure S6) that exhibited antagonistic activity against *C. albicans* by inhibiting its Y-H transition (Ricci et al., 2022; Figure 3). This bacterial action may aid in reducing GI colonization by *C. albicans.* Another example is when archaeal methanogens (*Methanomassiliicoccales*) keep TMAO levels in check by metabolizing the bacterial metabolite, TMA into methane, thus being protective of TMAO-mediated heart conditions (Gaci et al., 2014; Liu and Dai, 2020; Figure 3).

Bacterial-fungal interactions have been studied with their synergistic interactions being associated with conditions such as CD. *C. albicans* and two bacterial species, *Serratia marcescens* and *Escherichia coli* co-occurred in CD with microscopy analysis suggesting that the bacteria and fungus physically interacted in a mixed biofilm (Hoarau et al., 2016). *In vitro* studies indicated that these fungal and bacteria species formed a thicker mixed biofilm than any of the species could generate individually, thus together creating a stronger commensal niche (Hoarau et al., 2016; Figure 3). Another study conducted in IBD patients identified positive correlations between the decreased abundance of *S. cerevisiae* and the reduction of several bacterial genera, including *Bifidobacterium*, *Blautia*, *Roseburia*, and *Ruminococcus*. The overall number and the magnitude of fungal–bacterial associations were higher in UC, with specific interactions potentially involved in the inflammatory processes, which are characteristic of IBD (Matijašić et al., 2020).

*In vitro* studies have indicated that commensal bacteria may increase human norovirus (HNoV) infection, which is the leading cause of acute gastroenteritis. This interaction is believed to be brought about by the histo-blood group antigens (HBGA) which are complex terminal carbohydrates present on red blood cells and mucosal epithelial cells (Walker and Baldridge, 2019). HBGA are also present on certain commensal bacteria. HNoV capsids can directly bind to enterocyte HBGAs as well as those produced by bacteria such as *Enterobacter* sp. (Miura et al., 2013). This binding may facilitate the retention of the virus in the intestine and counter the movement of particles via peristalsis, thus helping virus get transcytosed across intestinal epithelial cells (via M cells) (Figure 3). As a result, HBGA-expressing bacteria are thought to enhance HNoV infection (Karst and Wobus, 2015).

Owing to the intricate interactions between microbial kingdoms which can have an overall effect on the host’s health and disease outcome, there is a compelling need to take into account all microbial kingdoms and their metabolites while assessing GI conditions. Additionally, these interactions are important to consider even during the treatment of microbial dysbiosis. Antibiotics employed to treat bacterial overgrowth can induce changes in the composition of the other microbial kingdoms (Sam et al., 2017). It has been well-demonstrated that broad-spectrum antibiotics and antibiotics specific to anaerobic bacteria, can cause differential effects on fungal susceptibility and predisposes patients to *C. albicans-*mediated GI infections (Sam et al., 2017). The use of broad-spectrum antibiotics that act on anaerobic bacteria was associated with increased yeast microbiota in the gut compared with antibiotics with poor anaerobic activity (Samonis et al., 1993). As a result, antibiotic treatment can lead to a shift in gut microbiota which is why inter-kingdom associations and the effect of treatments on interkingdom interactions must be considered while diagnosing and treating GI conditions.

## 9. Prospects for gut microbiome assessment

### 9.1. Interkingdom gut microbial and metabolite assessment

The gut microbial ecosystem hosts organisms of various kingdoms and their fine interplay can be implicated in host health and disease (Willis et al., 2019). Therefore, gut microbiome analysis must offer testing across the microbial kingdoms which will enable a better understanding of the gut ecosystem. Previous studies mainly focused on gut bacterial analysis as bacterial species were found in abundance in human microbiota (Lapiere and Richard, 2022). However, with the dawn of recent studies indicating the presence and crucial roles of other kingdoms housed in the gut, it is important to assess these microorganisms as well (Duerkop and Hooper, 2013; Rampelotto, 2013; Gaci et al., 2014; Yang et al., 2016; Chin et al., 2020).

Inter-kingdom interactions between the microorganisms highlight the need for assessing these commensal entities together (Samonis et al., 1993; Miura et al., 2013; Karst and Wobus, 2015; Hoarau et al., 2016; Sam et al., 2017; Walker and Baldridge, 2019; Willis et al., 2019; Lapiere and Richard, 2022; Ricci et al., 2022). Microorganisms through their metabolic activities produce metabolites that are involved in various gut functions (Qamar et al., 2001; Beaud et al., 2005; Sam et al., 2017; Mehmood et al., 2019; Chin et al., 2020; Wu X. et al., 2021; Lapiere and Richard, 2022). Gut dysbiosis causing an alteration in microbial species can also lead to altered gut-metabolite levels which can in turn affect host health (Binder, 2010; Joossens et al., 2011; Blais Lecours et al., 2014; Gevers et al., 2014; Zhang et al., 2015; Davis, 2016; Ghoshal et al., 2016; Ishaq et al., 2016; Ahmadmehrabi and Tang, 2017; Nagao-Kitamoto and Kamada, 2017; Ghavami et al., 2018; Chong et al., 2019; Kim et al., 2019; Liu and Dai, 2020; Matijašić et al., 2020; Novakovic et al., 2020; Silva et al., 2020; Xiao et al., 2021; Zhang S. et al., 2021; Caetano and Castelucci, 2022; Hao and Son-Nam, 2022; Khorsand et al., 2022; Rashed et al., 2022). Keeping this in mind, the assessment of microbial metabolites may also be of clinical significance. Moreover, inter-kingdom interactions take place via metabolites, thus gut metabolite analysis is very crucial (Sam et al., 2017; Lapiere and Richard, 2022). Microbial interkingdom assessment along with their metabolites poses a holistic approach toward understanding the delicate ecosystem of the gut.

### 9.2. Gut keystone species

Gut keystone species are defined as those species that have critical functions which help in maintaining the organization and diversity of the gut ecological communities. They have integral roles and maintain the gut ecosystem through biotic interactions with other community members (Trosvik and De Muinck, 2015). They are present in relatively low numbers and their gain or loss may have a profound influence on the gut ecosystem (Shetty et al., 2017). Generally, keystone species are a part of the common core, and their capabilities may include integral functions such as the breakdown of complex carbon sources to support the growth of the other core members (Shetty et al., 2017). They may also participate in regulating immune responses, interact with other taxa, maintain homeostasis of the intestinal tract, and affect the host’s long-term health (Ashman and Krishnamurthy, 2019).

The taxa including *Lactobacillus*, *Bifidobacterium*, *Eubacterium*, *Clostridia*, *Butyrivibrio*, *Roseburia*, *Akkermansia*, *Faecalibacterium*, *Bacillus*, *Prevotella*, *Lachnospiraceae*, *Ruminococcus*, *Oxalobacter*, and *Blautia* have been identified as keystone species (Ashman and Krishnamurthy, 2019). *Faecalibacterium*, *Bifidobacterium*, *Eubacterium*, *Butyrivibrio*, *Roseburia*, *Prevotella*, *Lachnospiraceae*, and *Blautia* help in the production of integral gut metabolites such as butyrate, formate, lactate, acetate and so on (Sagheddu et al., 2016; Shetty et al., 2017; Tamanai-Shacoori et al., 2017; Rodríguez Hernáez et al., 2018; Maturana and Cárdenas, 2021; Tudela et al., 2021). These metabolites contribute to important roles such as regulating the gut microbiota composition, maintaining the gut barrier integrity, aiding in the gut immune responses, and maintaining energy homeostasis (Liu et al., 2022). *Akkermansia* is a dominant mucin degrader in the gut (Shetty et al., 2017). *Bacillus* can also degrade mucin (Shetty et al., 2017).

*Ruminococcus* has amylolytic activity that degrades resistant starch, and this helps support the growth of other bacteria capable of utilizing glucose, maltose, panose, and isomaltose for growth (Shetty et al., 2017). *Lactobacillus* and *Bifidobacterium* exhibit microbial bile salt hydrolase functions that aid in the deconjugation of BA (Tudela et al., 2021). *Clostridia* populate specific regions in the intestinal mucosa in close association with intestinal cells, which enables them to participate in crucial functions such as modulating physiologic, metabolic, and immune processes in the gut (Lopetuso et al., 2013). *Oxalobacter*, via its obligate requirement for oxalate as a source of energy and cell carbon, helps in the degradation of oxalate. This helps prevent the formation of oxalate crystals leading to kidney stones (Stewart et al., 2004).

Owing to their critical roles and low abundance, keystones species represent a vulnerable point in the gut ecosystem. A minor perturbation in the prevalence of keystone species can have a large impact on the composition of their resident microbial consortia (Fisher and Mehta, 2014). This makes them excellent targets for gut microbiome-based interventions. Improving their levels would regain the integrity and stability of the ecological system, thereby improving gut health (Wu et al., 2022).

### 9.3. Immunoglobulin assessment for gut dysbiosis

The GI tract provides a compartmentalized interface between the gut microbial ecosystem and the bloodstream (Zeng et al., 2016). The gut microbiota induces local immune responses by stimulating the growth of intestinal epithelial cells (IECs) (Gutzeit et al., 2014). IECs play crucial roles in maintaining intestinal immunity as they eradicate pathogens while tolerating or fostering colonization by commensals and mutualists (Gutzeit et al., 2014). For this reason, the intestinal mucosal surfaces produce and secrete IgA to maintain host-microbiota homeostasis (Gutzeit et al., 2014). Secretory IgA (sIgA) is the predominant form of antigen-specific immunity present in the gut lumen that surveils commensals and mounts a response against enteric pathogens (Yang and Palm, 2020). This brings about a protective effect that separates commensal bacteria from the apical surface of IECs (Gutzeit et al., 2014). sIgA binds to bacteria to form a coating, which prevents the bacteria from adhering to the gut lining, thereby reducing their ability to cause diseases (Mantis and Forbes, 2010). Symbiont bacteria such as Lactobacillus and Dorea species undergo lesser sIgA coating as opposed to potential pathobionts in the gut (Mantis and Forbes, 2010).

Immunoglobulin G (IgG) is the most abundant antibody in peripheral blood, and it enters the intestinal lumen because of a leaky gut (DuPont et al., 2022). IgG coats intestinal microbiota to a much lesser extent than IgA. Moreover, IgG-mediated responses to microbiota generally occur during systemic inflammatory challenges. Systemic IgG has been seen to be produced against gut pathogens including *Trichuris trichiura, Entamoeba hystolitica, Salmonella*, as well as gut commensal bacteria, such as *Bacteroides fragilis* and *E. coli* (Vujkovic-Cvijin et al., 2022). The elevated IgG response confers protection against microbial invasion. Generally, mucosal IgA prevents symbiotic bacteria from translocating to extra-intestinal organs by blocking their access to epithelial receptors (Vujkovic-Cvijin et al., 2022), which avoids the IgG-mediated systemic response to symbiotic bacteria. Systemic IgG against a gut microbiota member may act as a marker for the identification of microorganisms with an enhanced ability to translocate across the intestinal membrane and cause diseases (Vujkovic-Cvijin et al., 2022). Given the roles of IgA and IgG in surveilling the gut microbiota, the assessment of their levels in circulation may help identify microorganisms with a greater ability to translocate and activate the immune system, which could potentially lead to diseases.

## 10. Effect of the gut microbiome on overall well-being

Although the gut is physically distant from the brain, it is known to affect the individual’s overall well-being. This effect could probably be mediated via the GBA through neural, hormonal, and immunological actions (Bull and Plummer, 2014). Several studies have demonstrated the association of gut dysbiosis with various features that profoundly affect the well-being of individuals. The commensal bacteria, *Actinobacteria* and *Proteobacteria* were increased while *Faecalibacterium*, *Bifidobacteria*, and *Lactobacilli* decreased in association with depression and anxiety (Li et al., 2018). Fatigue was associated with the increased abundance of *Enterococcus* and *Lactobacillus* and a reduction in *Escherichia coli* and *Bifidobacterium* populations (Li et al., 2018). Poor sleep correlated with an increase in *Lachnospiraceae*, *Blautia*, *Parasutterealla*, *Coprococcus*, and *Oribacterium*, and a reduction in *Bacteroidetes*, *Actinobacteria*, *Firmicutes*, *Corynebacterium*, *Sutterella*, and *Brevibacterium* (Smith et al., 2019). These bacterial species are known to contribute to the production of GABA and serotonin, neurotransmitters that aid in sleep; reduction in the bacterial species synthesizing the mentioned neurotransmitters will reduce their levels which will eventually affect sleep quality (Smith et al., 2019). As a result, bacterial species can affect the host’s well-being. With limited studies being carried out in other microbial kingdoms, the associations of these microbial entities with features of the host’s well-being are unclear. Nevertheless, a study showed that there was increased growth of the fungus, *C. albicans* in association with fatigue (Li et al., 2018). Given the profound effects of archaea, fungi, and viruses on host health and disease, microbial species belonging to these kingdoms could also be affecting the host’s well-being, and further research could elucidate the same.

## 11. Clinical applications of gut microbiome assessment

The association of the gut microbiome with host health has led its assessment to be a potential tool for disease prediction, diagnosis, and therapeutics. Studies have demonstrated the applicability of using gut microbiota composition and metabolite profiles in predicting future disease onset. Zheng et al. reported that the phylum *Bacteroidetes* was increased in coronary artery disease (CAD) conditions, making the phylum a microbial marker for distinguishing CAD patients from healthy controls (Zheng et al., 2020). Similarly, a study conducted by Leonard et al. (2021) revealed that the inflammatory bacteria, Dialister invisus, *Parabacteroides* sp., *Lachnospiraceae*, and the metabolites, tryptophan, serine, and threonine were increased in celiac patients. These patients also had lower levels of the anti-inflammatory bacteria, *Streptococcus thermophilus*, *Faecalibacterium prausnitzii*, and *Clostridium clostridioforme*. The alternations in the levels of pro-and anti-inflammatory bacteria indicate that in addition to gluten and genetic compatibility, the gut microbiome may also be contributing to the pathogenesis of celiac disease. Overall, these studies indicate that gut micro-organisms can arise as disease progression markers to identify hidden pathologies.

Gut microbial markers exhibit strong diagnosis potential and can be used along with standardized tests. In a study, Ren et al. (2018) established a successful diagnosis model for HCC via the identification of 30 microbial markers through a fivefold cross-validation on a random forest model. Differences in the gut metabolite profile were also observed with the decreased abundance of butyrate-producing bacteria in HCC patients (Ren et al., 2018). In a study by Deng et al. (2022) fecal assessment revealed the increased prevalence of gram-negative bacteria in cholangiocarcinoma (CCA) in comparison with HCC patients. Understanding the shifts in microbial populations for various diseases may help in distinguishing one condition from the other. Notable changes in specific gut micro-organisms may also aid in elucidating the contribution of the organisms in the pathogenesis of a given condition. General gut microbial or metabolic assessment presents as an inexpensive screening tool that could be used along with standardized tests. With increased attempts to establish specific profiles for a range of diseases, general gut microbial screening may gain momentum in non-specialty medical settings (Turjeman and Koren, 2022).

The association of the gut population with health and disease states suggests its implications in therapeutics as well. Microbial manipulation via supplementation can aid in the treatment for a range of diseases. Based on the microbial profile, personalized probiotics enable formulating bacterial or metabolite cocktails in a disease-specific manner intending to regain homeostasis (Turjeman and Koren, 2022). Moreover, gut microbiome assessment can also be used to predict host responsiveness to treatments. In the case of rectal cancer, patient response to neoadjuvant chemoradiotherapy (nCRT) was predicted with >73% accuracy in a validation cohort. This was done using 10 microbial taxa which were differentially expressed between responders and non-responders as predictors (Yi et al., 2021). Pre-screening patients’ microbiota to determine the success of certain treatments may become a standard practice in clinical settings. Gut microbial assessment can not only aid in determining the success of treatments, but microbial-based medication approaches could also reduce the unwanted effects of certain medications. A study by Chaput et al. (2017) was able to predict the metastatic melanoma patients who were more likely to develop colitis based on surveying their pre-treatment microbial profile. Another study showed that the microbiome of cancer patients who may gain weight following chemotherapy was different from that of those who will not. This indicates that gut microbial assessment can be used to anticipate and reduce unwanted side effects of therapeutics.

With the indispensable role of the gut microbiome and its metabolites in GI symptoms, GI and extra-GI conditions, and the host’s well-being at large, the assessment of gut micro-organisms and their metabolites across kingdoms could potentially become a major clinical tool in understanding host health and disease.

## 12. Conclusion

In this review, we discussed the prevalence of the bacteriome, mycobiome, archaeome, and virome as entities of the gut microbiome. The normal functioning of the gut microbiome is very important as it plays crucial roles in host metabolism. Gut dysbiosis is characterized by perturbations in microbial populations and can have implications not only on the gut but the overall health of the host. Gut microorganisms also produce metabolites that are important for several host metabolic functions and interkingdom microbial interactions. An alteration in microbial species can affect their respective metabolite concentrations which can lead to several health effects. We focus on the need for interkingdom microbial analysis and testing of their metabolites for holistic assessment of the gut that will aid in the understanding of gut dysbiosis. Additionally, serum sIgA and IgG levels may help identify commensals that can translocate through the gut and act as pathobionts by activating the immune system. As gut microorganisms have profound effects on human health and well-being, gut microbiome assessment can be an effective tool in understanding the pathophysiology of various diseases leading to more holistic treatments.

## Author contributions

HK, JR, and VJ: conception. HK, MP, JB, JG, VJ, KK, TW, KB, and JR: manuscript preparation. All authors contributed to the article and approved the submitted version.

## Glossary

GI: Gastrointestinal
GBA: Gut-brain axis
SCFAs: Short-chain fatty acids
BA: Bile acids
IBS: Irritable bowel syndrome
IBD: Inflammatory bowel diseases
CRC: Colorectal cancer
NAFLD: Non-alcoholic fatty liver disease
CVD: Cardiovascular disease
UC: Ulcerative colitis
CD: Crohn’s disease
IPA: Indole propionic acid
PXR: Pregnane X receptor
JAM: Junctional adhesion molecules
ZO-1: Zonulas occludens
AMPK: AMP-activated protein kinase
GPCR: G-protein-coupled receptors
IL: Interleukin
STAT-3: Signal transducer activator of transcription 3
T2D: Type 2 diabetes
HCC: Hepatocellular carcinoma
ALD: Alcoholic-liver disease
TMA: Trimethylamine
TMAO: Trimethylamine N-oxide
CRP: C-reactive protein
ASD: Autism spectrum disorder
BBB: Blood brain barrier
DCA: Deoxycholic acid
SIBO: Small intestinal bacterial overgrowth
BSH: Bile-salt hydrolase
IgA: Immunoglobulin A
Y-H: Yeast-to-hyphal
CNS: Central nervous system
IFN: Interferon
TNF: Tumor necrosis factor
NASH: Non-alcoholic steatohepatitis
RTT: Rett syndrome
TLR: Toll-like receptors
CYP7A1: Cholesterol 7α-hydroxylase
CYP27A1: Sterol-27-hydroxylase
CYP8B1: Sterol 12α-hydroxylase
CDCA: Chenodeoxycholic acid
CA: Cholic acid
FXR: Farnesoid X receptor
LCA: Lithocholic acid
GPBAR1: G protein-coupled bile acid receptor 1
FGF19: Fibroblast growth factor 19
GLP-1: Glucagon-like peptide 1
UDCA: Ursodeoxycholic acid
AhR: Aryl hydrocarbon receptor
Th: T-helper
AD: Alzheimer’s disease
5-HT: Serotonin
5-HT3R: Serotonin type 3 receptors
5-HT4R: Serotonin type 4 receptors
IBS-C: Constipation predominant IBS
ArAT: Aminotransferase
ILA: Indole lactate
IA: Indole acrylic acid
CKD: Chronic kidney disease
TPH: Tryptophan hydroxylase
SERT: Serotonin transporter
HDC: Histidine decarboxylase
HRH: Histamine receptor
ImP: Imidazole propionate
UrdA: Urocanate reductase
L-DOPA: Levodopa
DRD: Dopamine receptors
PAA: Phenylacetic acid
PAGln: Phenylacetylglutamine
PEA: Phenylethylamine
pCS: P-Cresol sulfate
GABA: γ-aminobutyric acid
CCK: Cholecystokinin
GABAR: GABA receptors
Lac-Phe: N-lactoyl-phenylalanine
3,4HPAA: 3,4-dihydroxyphenylacetic acid
EQs: Equols
DHR: Dihydroresveratrol
NAAs: N-acyl amino acids
S1PR4: Sphingosine-1-phosphate receptor 4
PTGIR: Prostaglandin I2 receptor
PTGER4: Prostaglandin E receptor 4
IECs: Intestinal epithelial cells
sIgA: Secretory immunoglobulin A
IgG: Immunoglobulin G
CAD: Coronary artery disease
CCA: Cholangiocarcinoma
nCRT: Neoadjuvant chemoradiotherapy
