# Supplementary figures and images for: Gut commensals and their metabolites in health and disease
Source: Front Microbiol. 2023 Nov 8;14:1244293. doi: 10.3389/fmicb.2023.1244293 (PMC10666787; doi:10.3389/fmicb.2023.1244293)

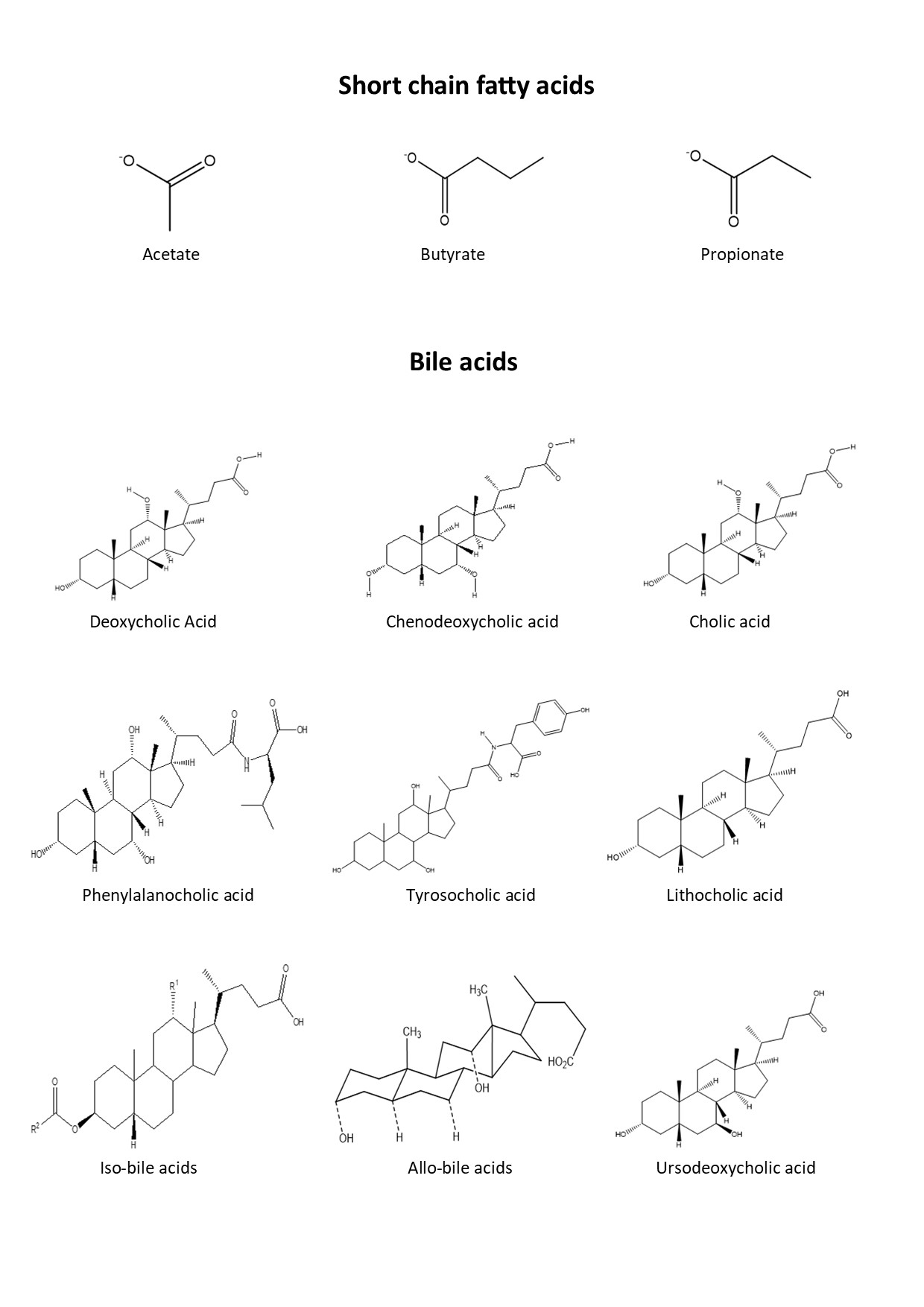

Supplement: Supplementary file 1 [file Data_Sheet_1.ZIP › SM, Figure - 1.jpg]

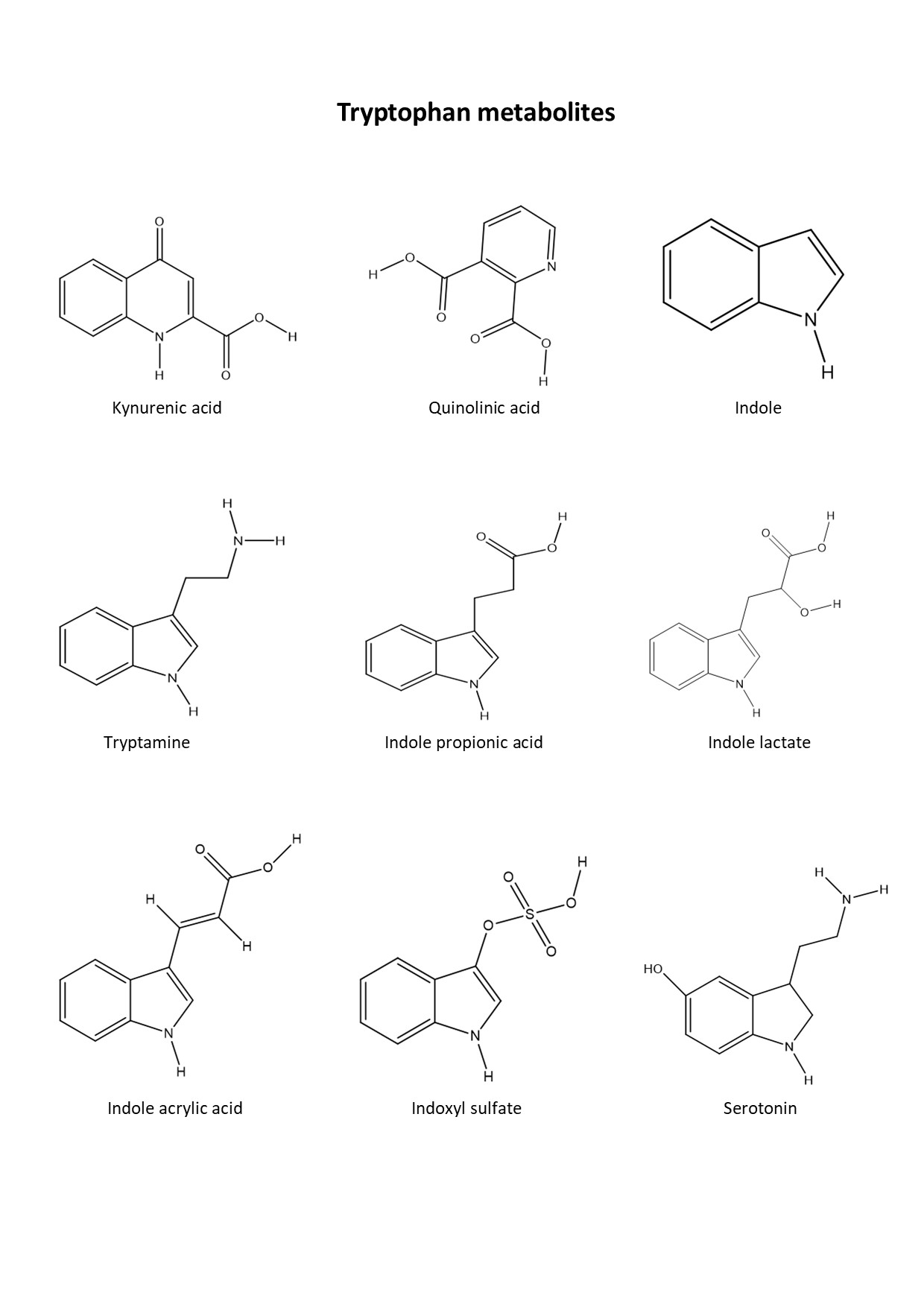

Supplement: Supplementary file 1 [file Data_Sheet_1.ZIP › SM, Figure - 2.jpg]

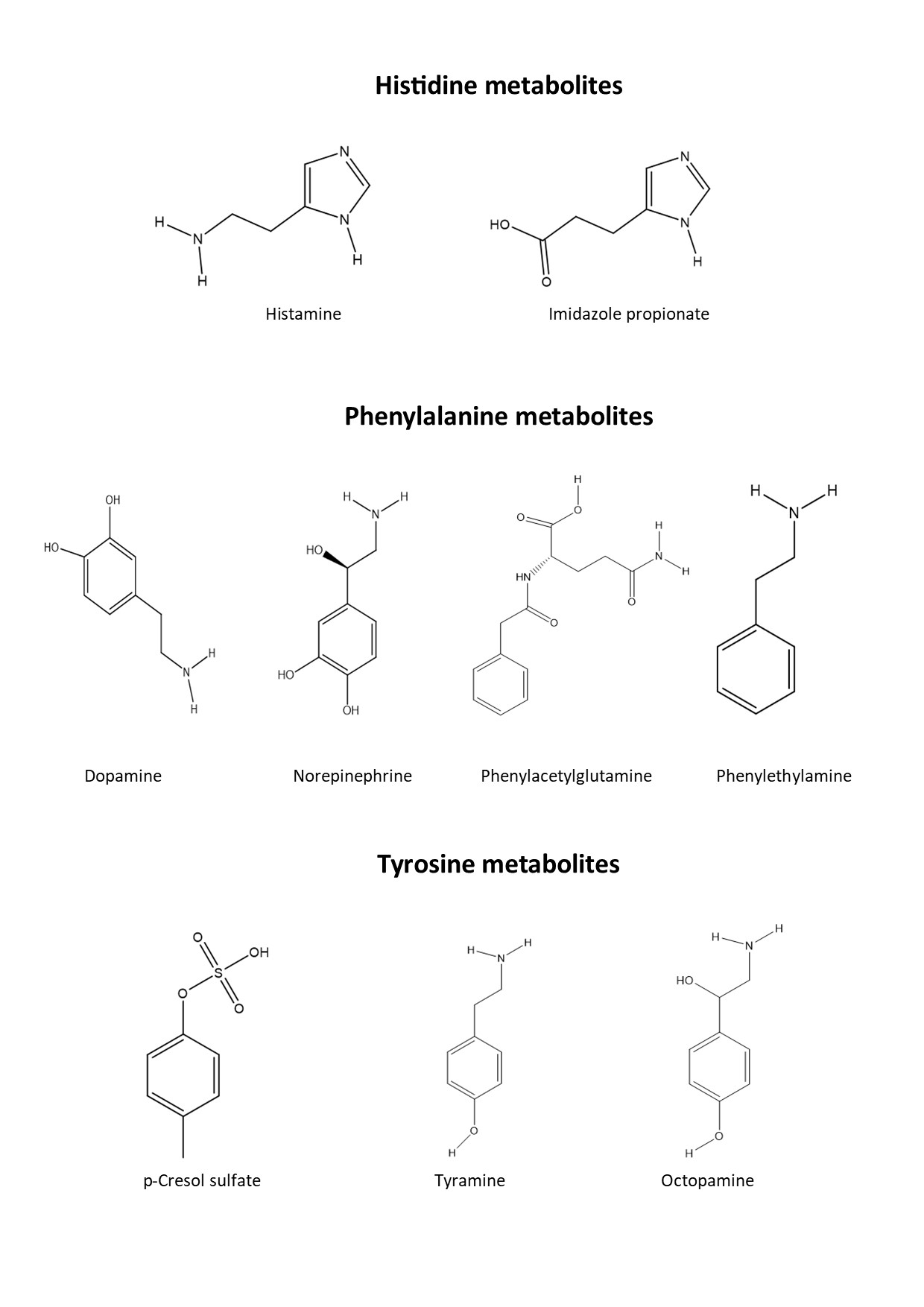

Supplement: Supplementary file 1 [file Data_Sheet_1.ZIP › SM, Figure - 3.jpg]

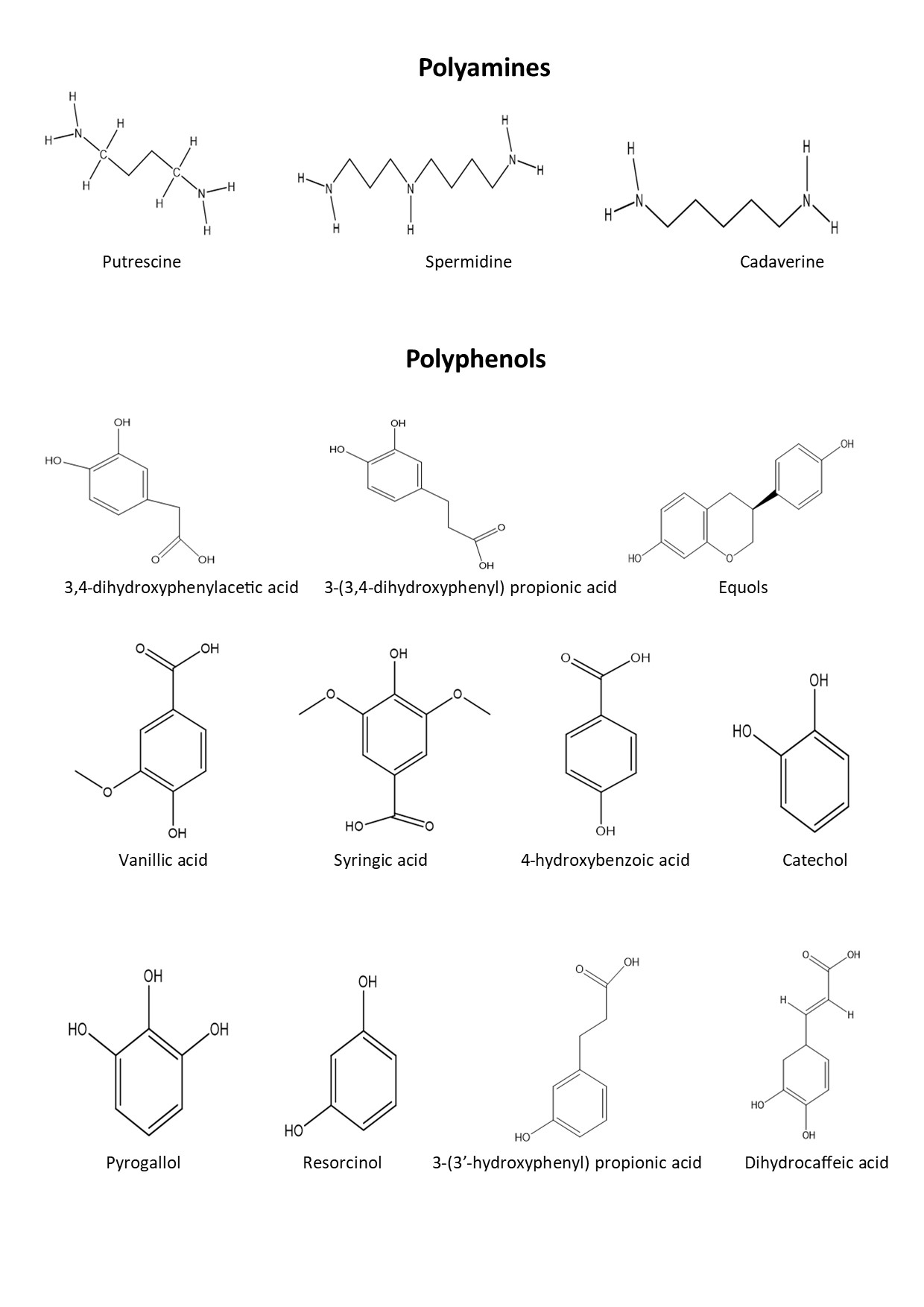

Supplement: Supplementary file 1 [file Data_Sheet_1.ZIP › SM, Figure - 4.jpg]

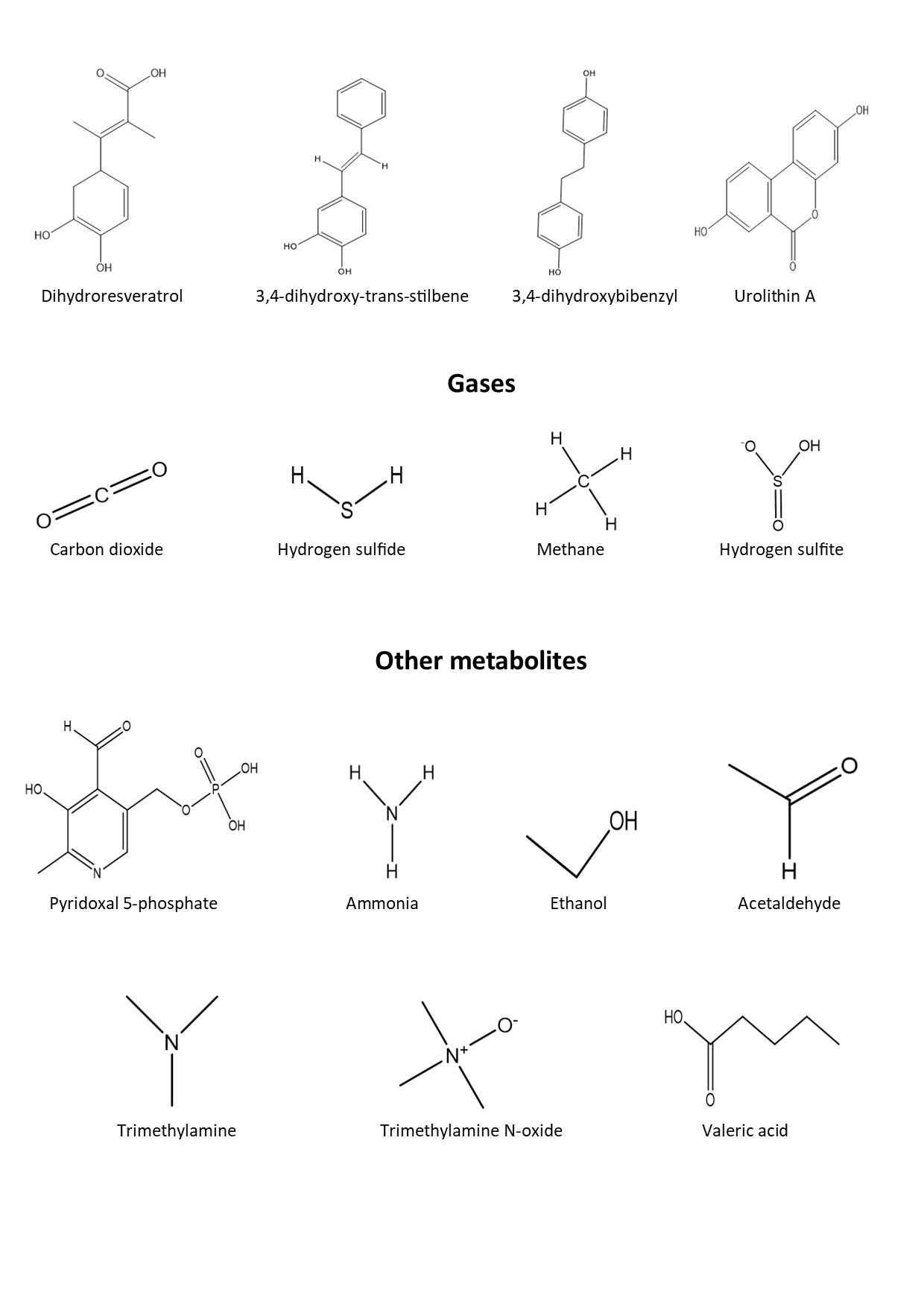

Supplement: Supplementary file 1 [file Data_Sheet_1.ZIP › SM, Figure - 5.jpg]

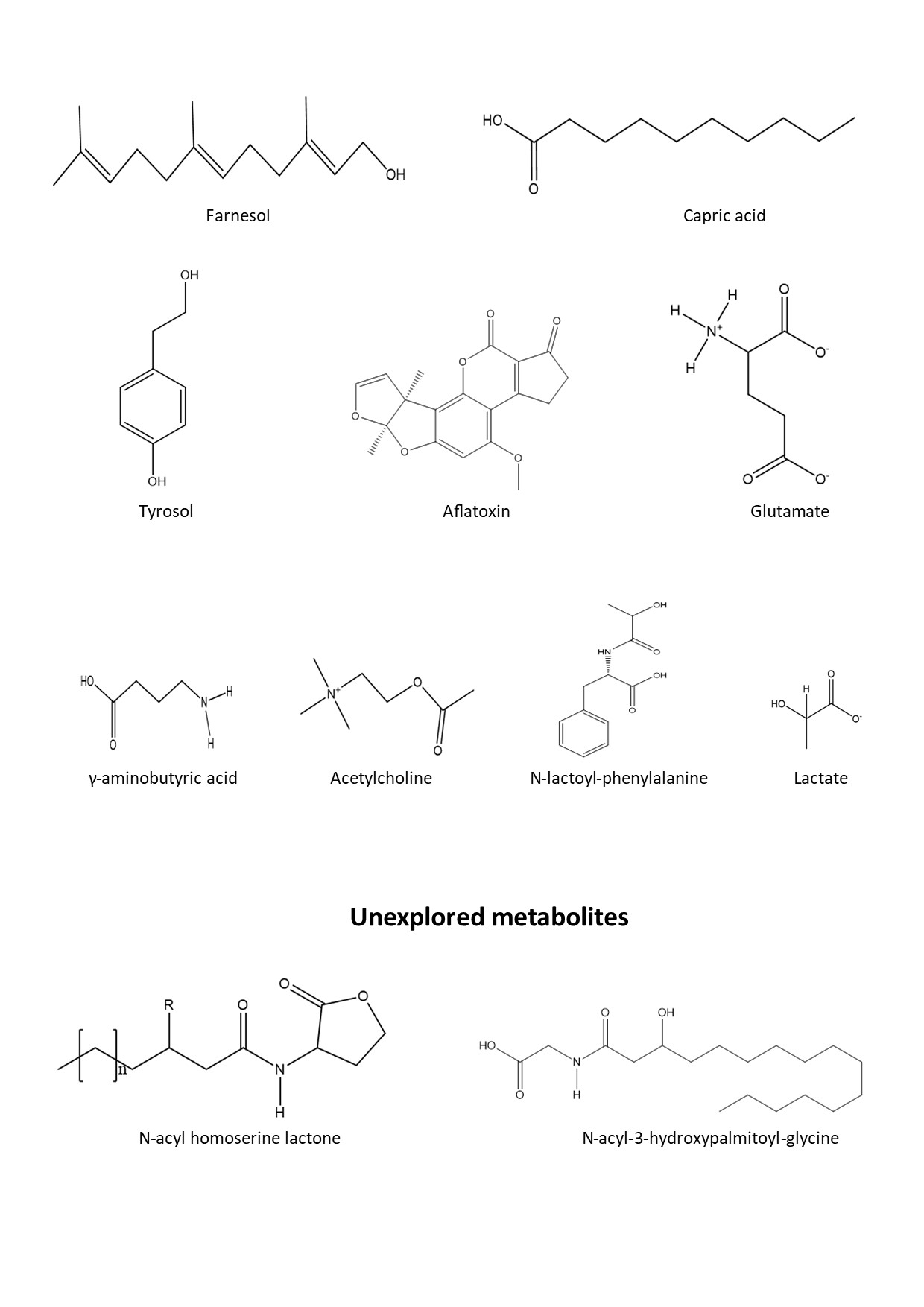

Supplement: Supplementary file 1 [file Data_Sheet_1.ZIP › SM, Figure - 6.jpg]

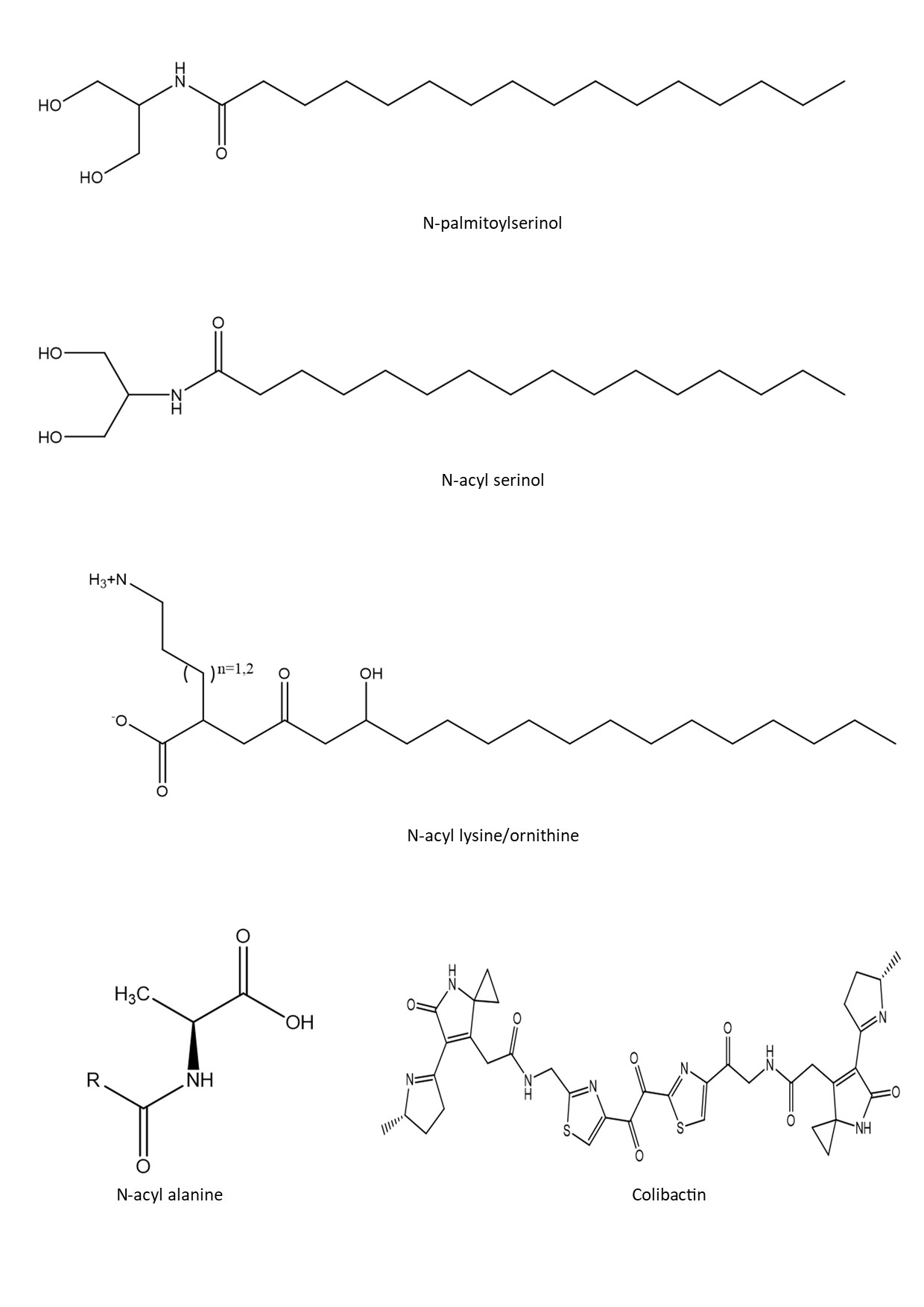

Supplement: Supplementary file 1 [file Data_Sheet_1.ZIP › SM, Figure - 7.jpg]
